# Image-guided treatment of mouse tumours with radioactive ion beams

**DOI:** 10.1038/s41567-025-02993-8

**Published:** 2025-08-19

**Authors:** Daria Boscolo, Giulio Lovatti, Olga Sokol, Tamara Vitacchio, Martina Moglioni, Francesco Evangelista, Emma Haettner, Walter Tinganelli, Christian Graeff, Uli Weber, Christoph Schuy, Munetaka Nitta, Daria Kostyleva, Sivaji Purushothaman, Peter G. Thirolf, Andreas Bückner, Jonathan Bortfeldt, Christoph Scheidenberger, Katia Parodi, Marco Durante

**Affiliations:** 1 GSI Helmholtzzentrum für Schwerionenforschung, Darmstadt, Germany; 2 Department of Medical Physics, Ludwig-Maximilians-Universität München, Munich, Germany; 3 Department of Electrical Engineering and Information Technology, Technische Universität Darmstadt, Darmstadt, Germany; 4 Life Science Engineering Faculty, Technische Hochschule Mittelhessen, Gießen, Germany; 5 Institute of Physics, Justus-Liebig-Universität Gießen, Gießen, Germany; 6 Helmholtz Research Academy Hesse for FAIR, GSI Helmholtz Center for Heavy Ion Research, Campus Giessen, Giessen, Germany; 7 Institute of Condensed Matter Physics, Technische Universität Darmstadt, Darmstadt, Germany; 8 Department of Physics ‘Ettore Pancini’, University Federico II, Naples, Italy

**Keywords:** Experimental nuclear physics, Applied physics, Computational biophysics, Biological physics, Imaging techniques

## Abstract

Charged particle therapy with protons or heavier ions is one of the most effective radiotherapy techniques, but uncertainties in the beam range can limit its efficacy. Radioactive ion beams are ideal for image-guided particle therapy because isotopes that undergo β^+^ decay can be visualized with positron emission tomography. This allows spatial localization of the particle distribution in vivo, which can be correlated with the expected dose deposition for online beam range verification. Here we report the successful treatment of a mouse osteosarcoma using a radioactive ^11^C-ion beam. The tumour was located in the neck, close to the spinal cord, where deviations of even a few millimetres in the beam range could lead to unintended dose deposition in the spine and radiation-induced myelopathy, an injury to the spinal cord. We achieved complete tumour control with the highest dose of 20 Gy while avoiding paralysis. Low-grade neurological side effects were correlated to the activity measured by positron emission tomography in the spine. The biological washout of the activity from the tumour volume was dependent on the dose, indicating a potential component of vascular damage at high doses. This experiment marks a step towards future clinical applications of radioactive ion beams.

## Abstract (web summary)

Particle therapy is subject to uncertainties in the range of the beam. In this study, tumours in the necks of mice were treated with radioactive ion beams, which enabled real-time verification of the beam range.

## Main

Nuclear physics methods have been instrumental in improving cancer radiotherapy using accelerated charged particles (protons or heavier ions). Charged nuclei exhibit a favourable depth–dose distribution in the human body due to the Bragg peak^1^. Therapy with accelerated ^12^C ions is currently being conducted at 17 centres worldwide^2^. Although more expensive than proton therapy, it offers biological advantages in addition to the physical benefits of the Bragg peak^3^. Particle therapy is, however, much more sensitive to uncertainties in the beam range than conventional X-rays, because of the high dose deposited in the Bragg peak^4,5^. Several techniques are available to monitor the beam range by exploiting the nuclear interactions of the ions in the tissue^6^, including positron emission tomography (PET)^7^. PET in carbon ion therapy exploits β^+^-emitting isotopes, such as ^11^C and ^10^C, produced by the nuclear fragmentation of the therapeutic stable ^12^C beam in the patient’s body. The method was extensively tested during the C-ion therapy pilot trial at the GSI Helmholtz Centre for Heavy Ion Research in Darmstadt (Germany)^8^, then at the Heidelberg Ion-Beam Therapy Center (HIT) in Heidelberg (Germany)^9^ and more recently at the National Center of Oncological Hadrontherapy (CNAO) in Pavia (Italy)^10^ and Heavy Ion Medical Machine (HIMM) in Wuwei (China)^11^. However, the counting rate from projectile fragments is low, the activity peak is shifted with respect to the Bragg peak because the particle range of the isotopic fragments depends on their mass (for example, the range of ^11^C is approximately 91% of the range of ^12^C at the same velocity), and the image analysis has been performed offline. Therefore, PET in ^12^C-ion therapy remains marginal and could not reduce the range uncertainty as desired (<1 mm)^7^.

Most of these problems can be overcome by using radioactive ion beams (RIB) rather than stable beams for therapy. RIB are generally acknowledged as the main tool to address the most important modern questions in nuclear physics, as they allow the study of nuclei at extreme conditions^12–14^. In cancer radiotherapy, RIB have the same biological effectiveness as the corresponding stable ion beams^15,16^ but increase the PET signal-to-noise ratio by approximately an order of magnitude, reduce the shift between the activity and dose peaks, and mitigate the washout image blur with short-lived isotopes (for example, ^10^C) and online acquisition^17,18^. The reduced uncertainty in range allows a shrinkage of the tumour margins around the clinical target volume (CTV), and this is expected to reduce toxicity for both serial or parallel organs at risk (OAR)^19^. Attempts to use RIB in cancer therapy started already in the 80s, during the heavy ion therapy pilot project at the Lawrence Berkeley Laboratory (CA, USA)^20^. However, these efforts were consistently hindered by the low intensities of the secondary beams produced by fragmentation of the primary ions used for therapy (for a historical review, see ref. ^21^). Modern high-intensity accelerators that can produce RIB with intensity sufficient for therapeutic treatments^22^ can be used to test PET-guided heavy ion treatments. One of these facilities is GSI/FAIR (Facility for Antiproton and Ion Research) in Darmstadt^23^, where we started the Biomedical Applications of Radioactive Ion Beams (BARB) project, aimed at performing the first in vivo tumour treatment with RIB^17^.

**Fig. 1:**
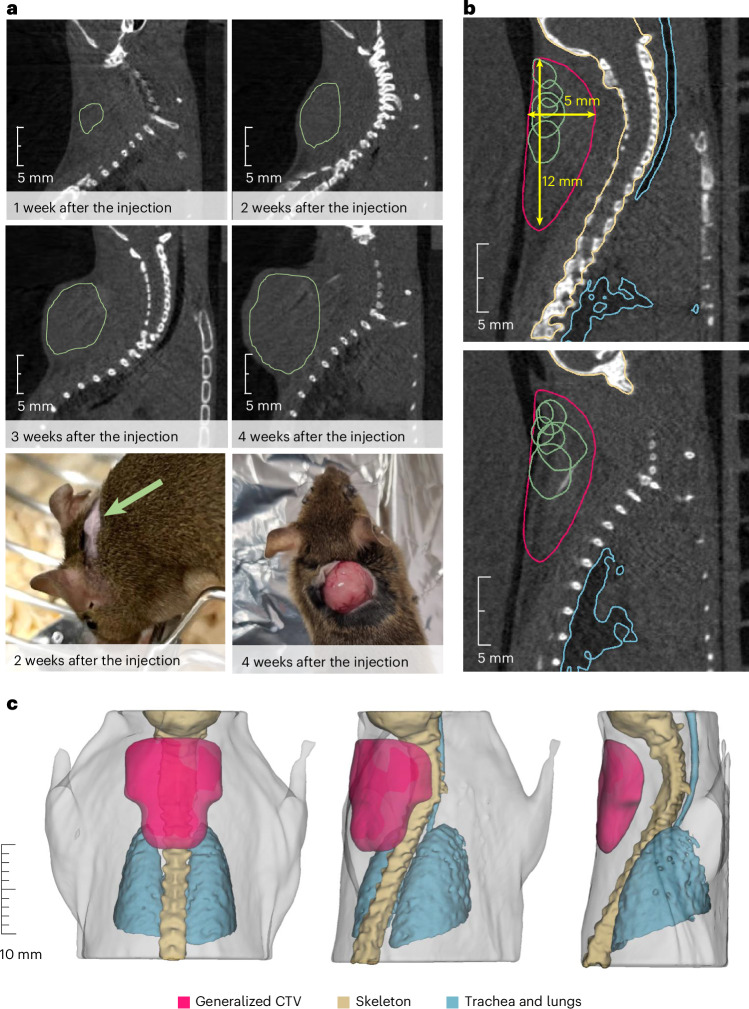
Mouse model and μCT. **a**, LM8 osteosarcoma as visible by eye or at the μCT at different times after cell inoculation. Green lines depict the GTV contours of the tumours. For the irradiation, a 2-week timepoint was chosen. **b**, Two slices of the CT depicting the contours of the individual tumour GTVs (green) near the OARs (spine and trachea with lungs, marked with yellow and blue, respectively). The generalized CTV contour (purple) was applied to all animals, covering all the possible GTV locations previously identified in the tumour induction study. Not all the GTVs are depicted here, as some of them were located on the different neighbouring CT slices. **c**, The CTV obtained from the contours of individual GTVs of tumours that grew in the animals used to establish and confirm the tumour model. To account for further biological variation, the resulting contour was smoothened and made symmetrical with respect to the spine. The CTV is depicted in purple; the mouse skeleton is shown in light yellow; the trachea and the lungs are shown in blue. The light-grey colour depicts the contours of the mouse body.

**Extended Data Fig. 1:**
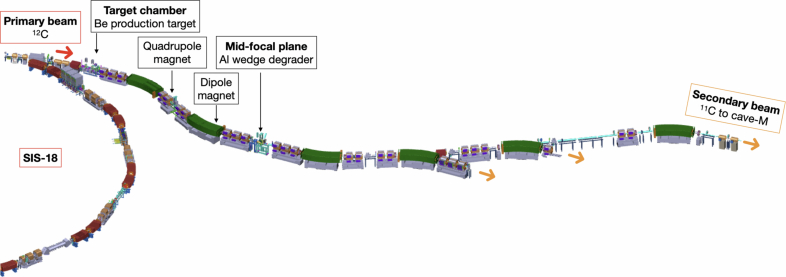
Fragment separator at GSI. A schematic view of the FRS is shown. A primary beam of ^12^C-ions from the SIS-18 synchrotron was incident on a beryllium target to produce ^11^C-ions, separated using the Bρ-ΔE-Bρ method. An achromatic degrader was placed at the mid-focal plane of the FRS. The FRS has three experimental branches for delivering the separated radioactive ion beam. The branch directed to Cave-M is indicated in the figure. The elements between the last dipole of the FRS and Cave-M belong to the high-energy beam transport line of GSI, designed for primary beams and therefore having smaller apertures than the FRS magnetic elements, leading to reduced transmission efficiency of secondary beams to the Cave-M. Figure courtesy of the DMU (Digital Mock Up) unit at GSI.

Within BARB, we have already reported the RIB imaging resolution in phantoms^24,25^ and transported the beam from the fragment separator (FRS) to the medical vault (Cave M, where animal experiments are possible) at the GSI accelerator facility^26^ (Extended Data Fig. 1). In Cave M, we then installed the portable small-animal in-beam PET scanner^27^, built by the Ludwig-Maximilian-University group in Munich for online range verification in preclinical particle therapy experiments in frames of the SIRMIO (Small animal proton Irradiator for Research in Molecular Image-guided radiation-Oncology) project. PET physics inherently requires ~30–60 s to accumulate sufficient statistics from radioactive decays for meaningful image updates, depending on the beam intensity. While not truly instantaneous, the data processing itself occurs on the millisecond scale, pushing the boundaries of in-beam PET monitoring. The SIRMIO PET scanner is based on 56 scintillator blocks of pixelated lutetium–yttrium oxyorthosilicate (LYSO) crystals. The crystals inside each detector block are arranged to provide a pyramidal-step shape to optimize the geometrical coverage in a spherical configuration^28^. Inside the detector it is possible to accommodate an anaesthetized mouse in vertical position, by using a custom three-dimensionallly (3D)-printed holder, for simultaneous irradiation and online PET imaging. The mouse model used in this study is a syngeneic LM8 osteosarcoma^29^ implanted in the C3H mouse neck. Osteosarcoma is a very radioresistant tumour^30^, and for this reason it is a typical candidate for treatment with accelerated ^12^C ions^31^. Figure 1a shows micro-computed tomography (μCT) images of the tumour growth after injection in the C3H mouse and the actual visible tumour in the neck. Figure 1b shows the contouring of the individual gross tumour volumes (GTVs) of the different mice used in the experiments. By summing up all the tumour profiles and smoothing the resulting outline, we have contoured a generalized CTV applied to all mice in this study (Fig. 1c; see the Methods for details). The proximity of the CTV to the spinal cord makes image guidance during treatment delivery a useful method to avoid radiation myelopathy^32–34^, a severe late effect of radiotherapy caused by white matter injury that can lead to motor deficits and paralysis^35^. Measured endpoints were tumour growth, spinal cord toxicity and washout rate of the radioactive signal from the tumour. We elected to use ^11^C projectiles even if our previous experiments^24,25^ show that the highest range-resolving power can be achieved with short-lived isotopes such as ^10^C or ^15^O. We preferred to use carbon, which is already used in many clinical facilities, rather than oxygen. Moreover, the intensity of isotopes that have lost two neutrons compared with the projectile in the FRS, such as ^10^C fragments from ^12^C primary beams, is too low for very high-dose (≥20 Gy) single-fraction treatment. The goal of the experiment was to use a ^11^C-ion radioactive beam to achieve full tumour control of a radioresistant tumour, such as osteosarcoma, proximal to an OAR, while maintaining low toxicity using online PET image guidance.

## The BARB beamline

**Fig. 2:**
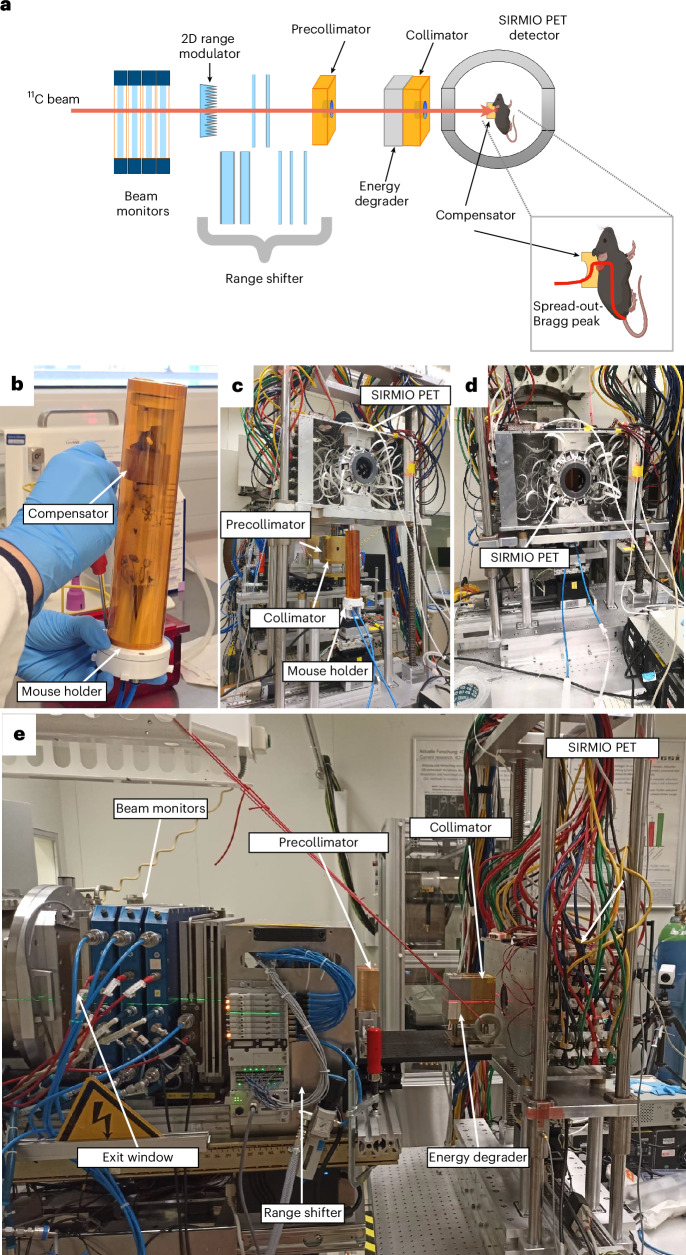
Experimental beamline. **a**, Drawing of the different elements along the experimental beamline. The mice were irradiated in a vertical position inside the SIRMIO PET scanner while a series of passive components shaped the beam to match the desired irradiation volume. In particular, calibrated large-plate ionization chambers were used as beam monitors for the pristine ^11^C beam. A 2D range modulator shaped the beam energy, creating a 1.2-cm SOBP in water. A range shifter and aluminium degraders were then used to adjust the beam range to approximately match the tumour position. Two brass collimators were used to reduce the lateral irradiation field and block parts of the beam that did not contribute to the target dose. Finally, a plastic mouse collar acting as a compensator was fixed to the mouse bed. It was designed to partially absorb the beam outside the CTV and shape the distal edge of the SOBP to match the target contour. **b**, SIRMIO animal holder with the anaesthesia tubes and a mouse in position. **c**, Animal holder aligned in the beamline while the SIRMIO PET is raised. **d**, The SIRMIO PET scanner is then lowered to surround the animal. **e**, Lateral view of the full beamline. Panel **a** created with BioRender.com.

Figure 2 shows the full BARB beamline prepared in Cave M at GSI along with photographs of different components. The secondary beam of ^11^C comes from the FRS^36^ (Extended Data Fig. 1). The primary intensity of the ^12^C-ion beam in the 18 Tm heavy ion synchrotron (Schwerionensynchrotron; SIS18) at 300 MeV u^−1^ was 1.6 × 10^10^ particles per spill, and the intensity of ^11^C ions in Cave M entrance was 2.5 × 10^6^ particles per spill. To maximize the online PET acquisition time, we used a short spill duration of 200 ms and a relatively low duty cycle with a repetition rate of 3 s (see Extended Data Table 1 for a summary of all the parameters).

**Extended Data Fig. 2:**
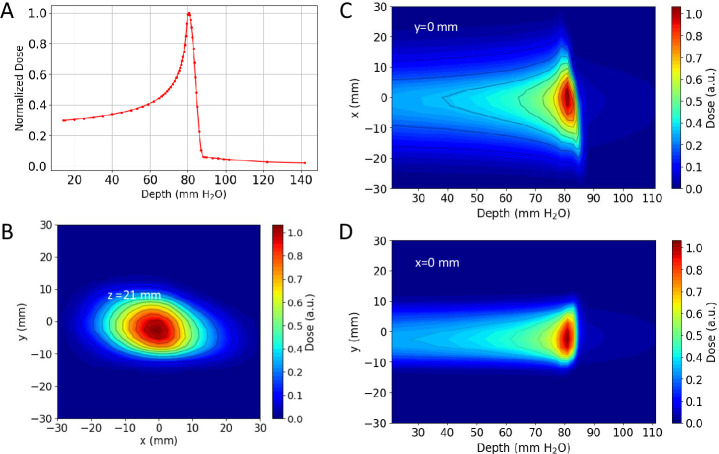
Dosimetry of the ^11^C beam. PEAKFINDER^TM^ measurements of the laterally integrated monoenergetic (pristine) beam depth dose profile in water. **b**. Water phantom measurement of the beam spot 2D dose distribution at the minimal water equivalent depth (*z* = 21 mm) inside the phantom. **c**, **d**. Water phantom measurements of the horizontal and vertical 2D dose distributions in the central plane along the beam direction respectively. Figures are normalized to their maximum values and contours represent iso-dose lines.

**Extended Data Fig. 3:**
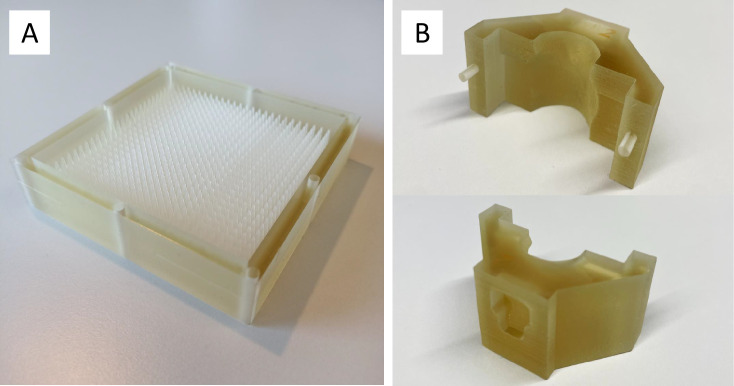
Cave M beamline components. The scheme of the beamline is shown in Fig. 2a. **a**. Range modulator used to generate the SOBP from a monoenergetic pencil beam scan. **b**. Plastic compensator to shape the lateral and distal edges of the irradiation field to the CTV. The length is 25 mm to shield the base of the skull and the rest of the animal spine. Top: inner side with an anatomical cut for fixing the animal neck area Bottom: outer side with a dimple corresponding to the distal edge of the CTV calculated in water-equivalent thickness. For a precise description of the compensator material see the section “Dosimetry” in the Supplementary Materials.

**Extended Data Fig. 4:**
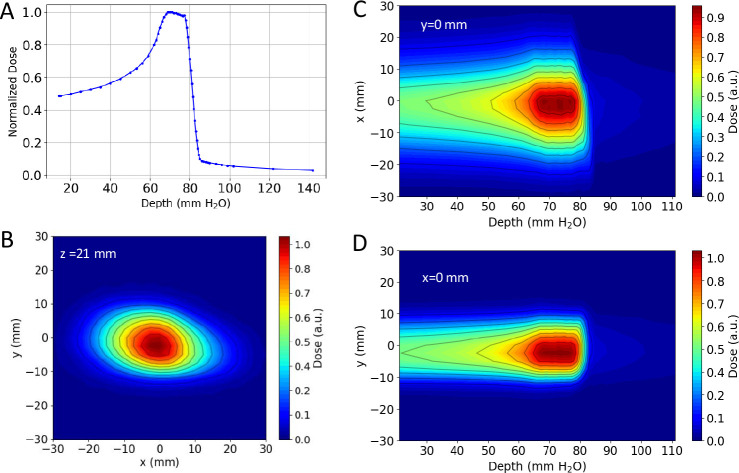
Dosimetry of the SOBP. **a**. PEAKFINDER^TM^ measurements of the laterally integrated SOBP depth dose profile in water. **b**. Water phantom measurement of the beam spot 2D SOBP dose distribution at the minimal water equivalent depth inside the phantom. **c**, **d**. Water phantom measurement of the horizontal and vertical SOBP 2D dose distributions in the central plane along the beam direction respectively. Figures are normalized to their maximum values and contours represent iso-dose lines.

A measured pristine ^11^C-ion Bragg curve is shown in Extended Data Fig. 2. To cover the full CTV, the pristine Bragg peak had to be widened to produce a spread-out Bragg peak (SOBP). The SOBP was formed by a 3D-printed range modulator (Extended Data Fig. 3a) from a two-dimensional (2D) scan of the monoenergetic pencil beam. The measured SOBP dose distribution is shown in Extended Data Fig. 4.

The distal field contour was modulated to the tumour CTV (Fig. 1b) by a 3D-printed plastic compensator collar (Extended Data Fig. 3b; from now on, simply indicated as ‘compensator’), also used as a holder for immobilization and positioning. We measured a dose rate around 1 Gy min^−1^ in the target volume covered by the SOBP. A total of 32 mice were irradiated with either a high (20 Gy) or low (5 Gy) tumour dose.

## PET activity

**Extended Data Fig. 5:**
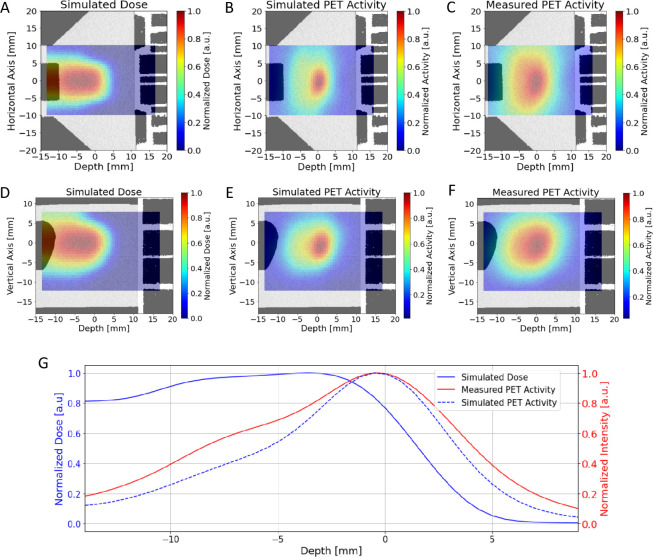
RIB imaging in phantoms. **a**. FLUKA simulation showing the expected ^11^C-ion dose (in Gy) distribution in the µCT of the plastic phantom in the axial view. Doses are normalized to one in the target region. **b**. Corresponding Monte Carlo simulation of the PET activity. **c**. Online SIRMIO PET image of the activity distribution deposited during ^11^C-irradiation overlaid on the same pre-treatment µCT used for the simulations. All the 2D images are integrated over the BEV aperture (±1 mm) in the plane transversing the beam direction and overlayed in the same axial µCT slice of the plastic phantom, which is shown in the background. **d**–**f**. The same as before, but in the sagittal view, with all the 2D images integrated on the same BEV aperture and overlayed in the same sagittal µCT slice of the plastic phantom, which is shown in the background. **g**. Simulated dose normalized to the target dose (blue), simulated PET activity (dashed blue), and measured (solid red) PET activity depth profiles, normalized to their maximum, along the z-axis direction, integrated over the BEV aperture (±1 mm) in the x-y plane transversing the beam direction.

In BARB, we use online PET imaging to monitor the RIB dose delivery. We first tested the consistency of the measurements and Monte Carlo simulations in plastic phantoms having the same shape and material composition as the compensator (Extended Data Fig. 3b). A Monte Carlo simulation of the ^11^C-beam interacting with a plastic phantom placed inside the SIRMIO PET scanner is shown in Extended Data Fig. 5, along with the measured PET image, both in transversal (Extended Data Fig. 5a–c) and lateral (Extended Data Fig. 5d–f) view. The Monte Carlo simulations include the full experimental set-up, the beam model and the PET signal formation in the detector, to account for the imaging process through the same reconstruction as for measured data. PET images are overlaid on the µCT scan of the irradiated phantom. In Extended Data Fig. 5g, we show the simulated dose, activity and the measured PET activity profiles along the *z*-axis direction, integrated on the beam’s eye view (BEV) aperture (±1 mm) in the *x*–*y* plane transversing the beam direction. We observed a good agreement between the simulations and experimental data, particularly in the peak region, where the PET activity peak aligns with the 80% SOBP dose fall-off. This supports the feasibility of the system for the in vivo experimental campaign. Differences in the measured and simulated activity profiles can be attributed to factors that degrade the measured PET signal, such as statistics, secondary radiation background and detector sensitivity, as well as to uncertainties in the Monte Carlo model and in the μCT calibration. These factors contribute to a higher intensity of the measured PET signal at the target entrance and a wider distribution compared with simulations.

**Fig. 3:**
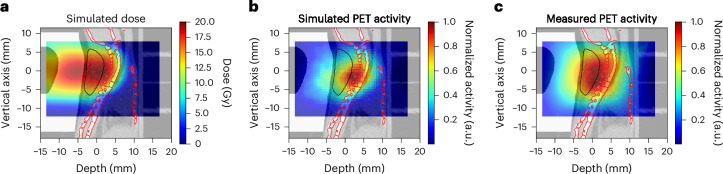
PET imaging in mouse. **a**, FLUKA simulation showing the expected ^11^C-ion dose (in Gy) distribution in the µCT of the mouse in the sagittal view. Doses are normalized to the planned target dose. **b**, Corresponding Monte Carlo simulation of the PET activity. **c**, Online SIRMIO PET image of the positron activity distribution deposited during ^11^C irradiation overlaid on the same pre-treatment µCT used for the simulations. All 2D distributions are overlaid on the same sagittal µCT slice of the same animal, which is shown in the background. The generalized CTV contour (Fig. 1) is highlighted with a black line, while the spine (OAR) contour is marked in red. All the images are integrated on the BEV aperture (±1 mm) in the *x* (axial) plane transversing the beam direction.

The PET activity in a mouse bearing the LM8 tumour is shown in Fig. 3 in sagittal view. We show the Monte Carlo simulation of the expected ^11^C-ion dose (in Gy) distribution calculated on the µCT of a mouse irradiated during the experiment (Fig. 3a) and the corresponding simulated PET activity (Fig. 3b). In Fig. 3c, we show the PET image acquired during the experiment overlaid on the same pretreatment µCT used for the simulations. All other measured PET images for the different mice irradiated with ^11^C-ion beam are shown in Supplementary Fig. 1. Supplementary Video 1 shows the build-up of the measured PET signal over the course of irradiation, in both sagittal and transversal views.

**Fig. 4:**
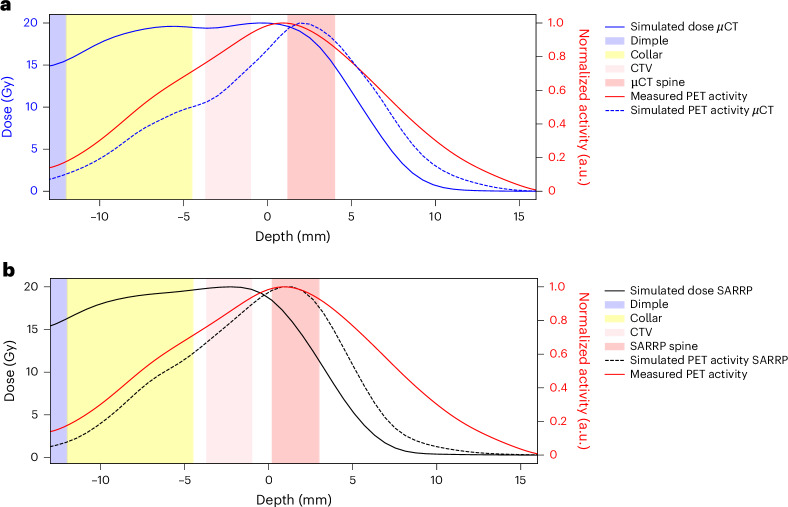
Activity profiles in mice. **a**, For the same mouse in Fig. 3, we show the *z*-axis depth profiles of the simulated dose (normalized to the target dose; blue), the simulated PET activity (dashed blue) and the measured (solid red) PET activity profiles, normalized to their maximum and laterally integrated on the BEV aperture (±1 mm) in the *x*–*y* plane orthogonal to the beam direction. The CTV and µCT spine regions are highlighted by pink and red bands, respectively, while the compensator is depicted in yellow and the dimple (Extended Data Fig. 3b) in light blue. **b**, Comparison between simulated dose and PET activity profiles for a mouse analysed at the CBCT in the SARRP in vertical position. The plot shows the *z*-axis depth profiles of the simulated dose (normalized to the target dose; solid black), the simulated PET activity (dashed black), and the measured (solid red) PET activity profiles, normalized to their maximum and laterally integrated on the BEV aperture (±1 mm) in the *x*–*y* plane orthogonal to the beam direction. Although the mouse is not the same as in **a**, we observed that switching to the vertical position consistently induces the same anatomical change in all animals. Therefore, we overlaid the measured PET activity profile of the mouse from Fig. 3 in red. As in **a**, The CTV and SARRP spine regions are highlighted by pink and red bands, respectively, while the compensator is depicted in yellow and the dimple (Extended Data Fig. 3b) in light blue.

**Extended Data Fig. 6:**
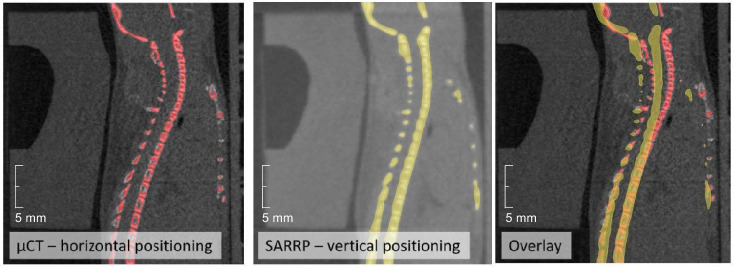
Mouse CT. Comparison of a scans with the μCT (left) and the SARRP CBCT (middle) for the same mouse. Red color depicts the contour of the mouse skeleton segmented from the μCT, and the yellow one from SARRP. The two scans are superimposed in the image on the left to highlight the small differences in the spine position.

Figure 4a shows the *z*-axis profiles corresponding to Fig. 3 integrated over the BEV aperture (±1 mm) in the *x*–*y* plane perpendicular to the beam direction. It can be noted that a shift of about 1 mm is observed between measured and simulated activity distributions. During the experiment, all PET images were overlaid on the corresponding pretreatment µCT scans of each mouse, acquired in horizontal position. However, actual irradiations were performed with mice in vertical position (Fig. 2), which may induce some anatomical changes. We conducted additional experiments to compare imaging in horizontal and vertical positions using the cone-beam CT (CBCT) of the Small Animal Radiation Research Platform (SARRP) installed in Cave M. Although the resolution of the CBCT is lower than that of the µCT, Extended Data Fig. 6 reveals small but consistent differences in spine curvature when the animals are placed in a vertical orientation. By including this anatomical shift in the simulation, the Monte Carlo calculation accurately reproduces the position of the measured activity peak (Fig. 4b). As for the plastic phantom measurements (Extended Data Fig. 5g), now the activity peak approximately aligns with the SOBP 80% dose fall-off. Residual discrepancies in the shape of the measured and simulated profiles are similar to those described above for the phantom image, plus additional uncertainties such as animal repositioning and non-homogeneous target composition, particularly the dimple in the compensator as well as different densities of the target (bone, hairs, fat, skin and so on).

**Extended Data Fig. 7:**
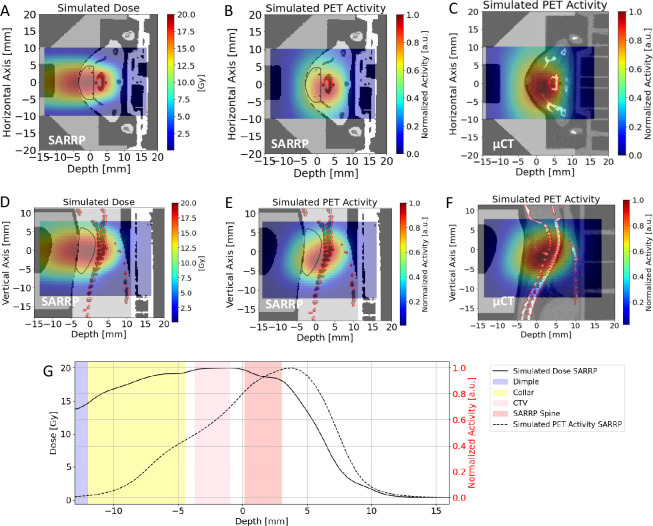
Simulation of PET imaging of a RIB damaging the spinal cord. **a**. FLUKA simulation showing a hypothetical case of a^11^C-ion SOBP dose (in Gy) distribution damaging the spinal cord for a vertical CBCT SARRP scan of a mouse in the axial view (shown in the background). Doses are normalized to the planned target dose. **b**. Corresponding Monte Carlo simulation of the PET activity as should have been seen with the SIRMIO PET scanner, overlaid on the same axial SARRP slice for the same mouse (displayed in the background). **c**. The activity simulation shown in **b** is now overlaid on the µCT scan (in the axial view, shown in the background) of the same mouse to illustrate what we would have seen online in case of RIB range exceeding the target distal position. The generalized CTV contour (Fig. 1) is highlighted with a black line, while the OAR contour is marked in red. All the images are integrated over the BEV aperture in the y-plane transversing the beam direction. In panels **d**, **e**, the same sagittal SARRP slice of the mouse is shown in the background of the 2D distributions. In panel **f**, the sagittal view of the µCT scan is displayed in the background. **g**. The corresponding simulated dose normalized to the target dose (solid black) and the simulated PET activity (dashed black) depth profiles, normalized to their maximum, along the z-axis direction, integrated over the BEV aperture in the x-y plane transversing the beam direction. The CTV and spine regions are highlighted by pink and red bands, respectively, while the compensator is depicted in yellow and the dimple in light blue. The plot shows that an online image as depicted in panels **c** and **f**, never actually observed in our experiments, would have required the insertion of a range shifter to reduce the range during the irradiation.

As the tumours were growing very close to the spinal cord, the online PET image was used especially in the first minutes of the irradiation to check that the SOPB was not covering the spine. Extended Data Fig. 7 shows a Monte Carlo simulation of the dose and corresponding predicted activity for an SOBP extending into the spinal cord, representing a case where the range is longer than expected. The simulation is superimposed on both μCT (Extended Data Fig. 7c–f) and CBCT (Extended Data Fig. 7b–e) images of the same mouse, in transversal (Extended Data Fig. 7a–c) and sagittal (Extended Data Fig. 7d–f) view. The images in Extended Data Fig. 7c–f were never observed in any animal (Supplementary Fig. 1). They would have meant doses as shown in Extended Data Fig. 7a–d, and unacceptable profiles as shown in Extended Data Fig. 7g. Therefore, no range correction by adjusting the degrader thickness (Fig. 2) had to be applied, even if the tumour was almost leaning on the spine.

## Tumour control

**Fig. 5:**
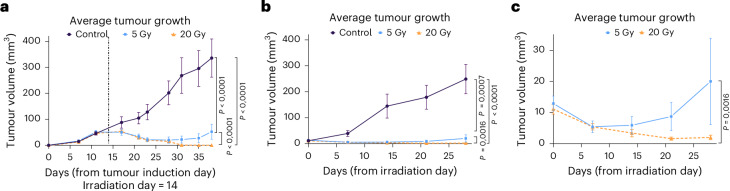
Tumour growth. **a**, Average tumour volumes calculated from 2D caliper measurements of the visible tumour (Methods) for the 0 Gy control group (*n* = 27 animals, purple line and circle symbols), 5 Gy group (*n* = 6 animals, light-blue line and square symbols) and 20 Gy group (*n* = 22 animals, orange line and triangle symbols). Over the course of observation period, 12 animals from the 0 Gy group had to be euthanized when the permitted tumour burden was reached. The vertical dashed line corresponds to the timepoint of irradiation. **b**, Measurements of the volumes using μCT. Data are more precise than caliper measurements, but they are less frequent than external measurements. **c**, Zoom of the data points for irradiated groups shows the recurrence of the tumour irradiated with 5 Gy. Bars are standard errors of the mean values of the different animals. For every type of tumour measurement data, a two-way analysis of variance was performed (GraphPad Prism version 10.5.0 (774)) to estimate the differences in the impact of different radiation doses on the tumour growth dynamics. All tests were two-sided, and effect sizes were not computed.

Tumour sizes of irradiated and control tumour-bearing animals were measured for 4 weeks after the day of irradiation using a caliper or μCT. Results shown in Fig. 5 demonstrate complete tumour control after 20 Gy and prolonged tumour growth delay after 5 Gy, with evidence of recurrence after 2 weeks. The data are compatible with a complete coverage of the tumour target for all animals in the ^11^C-beam treatment. Recurrence at the lower dose is expected considering the high radioresistance of the osteosarcoma.

## Toxicity

Skin toxicity scoring in the tumour-bearing controls was complicated by the growth of the tumour that caused superficial lesions (grade 4). In irradiated animals, all of them bearing small tumours after irradiation, skin toxicity was induced by radiation as shown in Supplementary Fig. 2. No irradiated animals showed skin toxicity grades >3.

**Extended Data Fig. 8:**
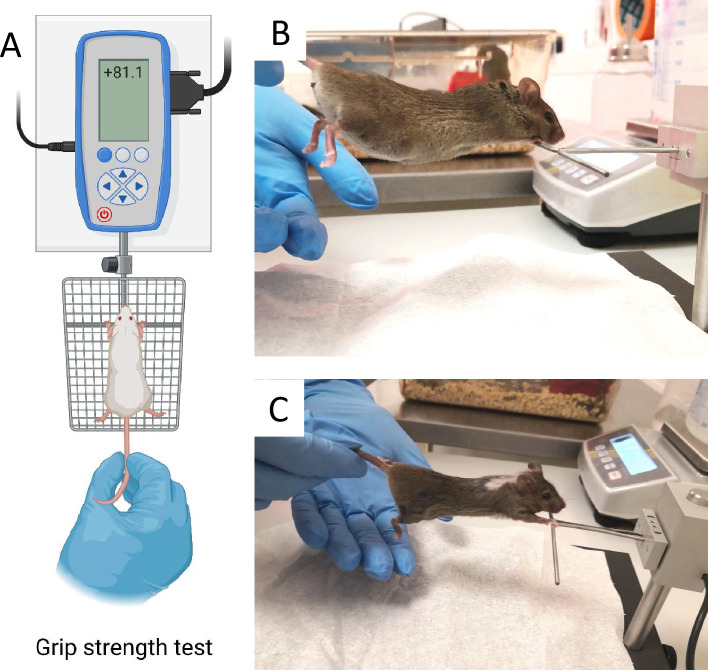
Grip strength test used to estimate the cervical myelopathy. **a**. In this quantitative test the animal is pulled by the tail and the strength of the grip on the bar is measured in newton (N). **b**. Control mouse during the test (lateral view). **c**. 20 Gy irradiated mouse during the test. The area behind the head is shaved prior to the tumor inoculation, and after the irradiation only some white fur grows back. Panel **a** created with BioRender.com.

**Extended Data Fig. 9:**
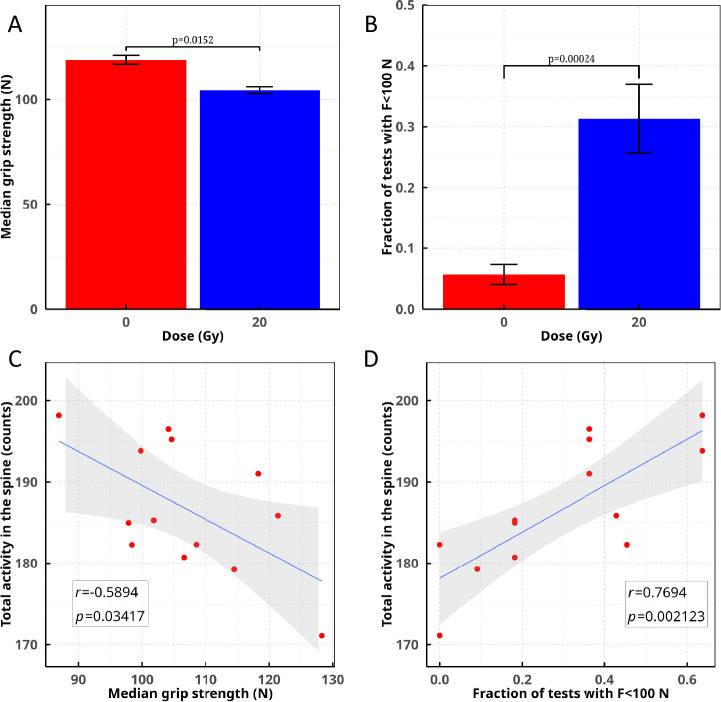
Toxicity vs. activity in the spinal cord. Animals exposed to ^11^C-ions shows a lower forelimb strength in the grip test (Extended Data Fig. 8) compared to controls. Individual data are shown in Supplementary Fig. 3. Data for irradiated animals were only considered from week 6, assuming no radiation effect in the first month post-irradiation. **a**. Median grip strength values in the control (8 animals) and irradiated (13 animals) groups. Bars are standard errors of the median values ($=1.2533\bullet \sigma /\sqrt{n}$). Median strength in the control group is significantly higher than in the irradiated group (Mood’s median test, *p* = 0.0152). **b**. Fraction of time points (Supplementary Fig. 3) where the measured peak force F was lower than 100 N. Bars are standard errors of the mean. The fraction is significantly higher in irradiated animals (Mann-Whitney test, *p* = 0.00024). **c**. Correlation between median grip strength values in single irradiated animals and total PET counts in the spinal cord. **d**. Correlation between fraction of tests with F < 100 N in individual irradiated animals and total PET counts in the spinal cord. Each point represents a single animal. The counts in the spinal cord are normalized to the total number of counts observed in the whole image reconstructed with the OSEM method. The grey area is the 95% CI around the regression line, *r* is the correlation coefficient and significance of the correlation was evaluated by Pearson’s test both in **c** and **d** panels. Plots produced with R v.4.2.1 ggplot package.

As the tumour was located very close to the cervical area of the spinal cord, the primary expected toxicity from high-dose exposure was radiation-induced myelopathy, as observed in previous experiments in mice^32–34^ or rats^37–40^. However, none of the animals exposed to ^11^C ions presented severe morbidity such as forelimb paralysis or pronounced kyphosis. The lack of severe toxicity demonstrates that the spine was not exposed to high doses, as observed with the PET measurement (compare Fig. 3 and Supplementary Fig. 1 with Extended Data Fig. 7). However, because the tumour is adjacent to the spine, some activity was inevitably observed in the spinal cord, located in the dose fall-off region (Fig. 4). We checked the impact of this residual dose on low-grade toxicity by measuring grip strength performance, a common test to assess cervical spinal injury^41^ (Extended Data Fig. 8). All results of the biweekly grip strength performance for individual mice are reported in Supplementary Fig. 3. A wide interindividual variability is noted in these curves. However, by pooling the data in Extended Data Fig. 9a,b, we show that the strength of the mice is reduced after irradiation compared with controls, indicative of a minor deficit in neuromuscular function. Correlation of integral PET counts in the spine with individual grip performance is shown in Extended Data Fig. 9c,d. Despite the wide scatter in the grip test data, there is a significant correlation between activity in the spine and decreased mouse forelimb strength. In this figure, the activity is measured on the μCT images of the irradiated animal. As shown in Supplementary Fig. 4, the anatomical changes due to repositioning (Extended Data Fig. 5) result in less than a 5% difference in activity counts and therefore do not affect the correlation shown in Extended Data Fig. 9c,d. We therefore demonstrate that the activity map of the radioactive therapeutic beams predicts toxicity in the OAR.

## Washout

The activity in a plastic target decreases after irradiation because of the physical decay of the ^11^C (*T*_1/2_ = 20.34 min) projectile. Additional positron emitters in the target are ^10^C and ^15^O. They are not beam contaminants but arise from nuclear fragmentation during tissue irradiation with the ^11^C beam: ^10^C is produced primarily by the beam’s fragmentation upon interacting with the target, while ^15^O is generated solely from tissue fragmentation (with soft tissue being ~65–75% oxygen). Their low production cross-sections (40–70 mb)^42^ yield abundances about two orders of magnitude lower than those of ^11^C. Moreover, the positron activity profile from tissue fragmentation drops near the Bragg peak—rendering ^15^O negligible (despite its *T*_1/2_ = 2.04 min) and allowing the distinct impact of ^10^C (*T*_1/2_ = 19 s) to be isolated from the ^11^C signal and biological washout effects.

**Fig. 6:**
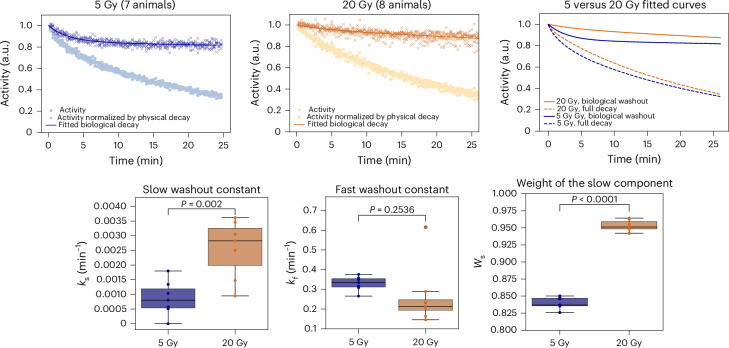
Radioactive washout. Top: individual activity data recorded after the end of irradiation in Supplementary Fig. 5 are grouped (left: 5 Gy (*n* = 7 animals), middle: 20 Gy (n = 8 animals), right: comparison of fit functions assuming the physics decay of the beam containing 96% ^11^C, 3% ^10^C and 0.5% ^15^O ions). Filled circles represent the total measured activity while the crosses correspond to the activity normalized for a physical decay. As a fit function, a double-exponential decay function was chosen over a single-exponential decay following the results of the *F* test (ratio of the fit *χ*^2^ with one or two parameters) with number of degrees of freedom d.f. >100. *F*(5 Gy) = 1126 (≫1), *F*(20 Gy) = 63 (≫1). The fit functions are depicted separately on the top left. Orange and blue lines are the fits of the 20 Gy and 5 Gy data, respectively. Dashed lines correspond to the fit of the full decay data, while the solid lines represent the fits of the biological washout data only. Bottom: double-exponential decay rates (*k*_s_ for the slow washout constant and *k*_f_ for the fast washout constant) and the weight of the slow component (*W*_s_) from equation (1) for 5 Gy and 20 Gy. Box plots display the median (line), interquartile range (box spanning the 25th–75th percentiles) and whiskers extending to the furthest data points within 1.5× the interquartile range. Circles display the individual data points, and colours represent treatment groups (blue for 5 Gy, orange for 20 Gy). Significance of the differences was assessed by two-sided unpaired *t*-test assuming equal variances. Effect size and confidence intervals were not computed. Data visualization and analysis were performed in Python 3.10 using the Seaborn and SciPy libraries.

We have previously modelled the radioactive decay with exponential functions that include all fragments produced^25^. In this experiment, the radioactive decay is overlapped with an unknown biological decay due to the blood flow in the tumour that removes the radioactive isotopes from the site of decay. The degree of vascularization in our tumour model was estimated by perfusion to opacify microvasculature structure in μCT. Supplementary Video 2 shows that our osteosarcoma in the neck is highly vascularized, so a strong biological washout is expected. The washout data for all animals are reported in Supplementary Fig. 5. Studies in Japan in a rat glioma model point to a double-exponential model for the biological washout^43^, which was also applicable to our data based on the results of the Fisher’s test on fitting parameters. We therefore used the following equation to fit the activity data measured after the irradiation was stopped:

1 $$A\left(t\right)={A}_{{{\mathrm{phys}}}}\times {A}_{{{\mathrm{bio}}}}={A}_{0}{\sum }_{i}{w}_{i}{{\mathrm{e}}}^{\frac{-\mathrm{ln}2}{{T}_{1/2i}}t}\times \left[{W}_{{\mathrm{s}}}{{\mathrm{e}}}^{{-k}_{{\mathrm{s}}}t}+(1-{W}_{{\mathrm{s}}}){{\mathrm{e}}}^{{-k}_{{\mathrm{f}}}t}\right],$$

where *A*_0_ is the activity at the end of the irradiation, *W*_s_ is the relative weight of the slow component, and *k*_s_ and *k*_f_ are the slow and fast time constants, respectively. *T*_1/2*i*_ is the half-life of the *i*th contributing radioisotope and *w*_*i*_ is its fraction in the total number of fragments. Based on the FLUKA Monte Carlo simulation, we have considered 96% ^11^C, 3% ^10^C and 0.5% ^15^O ions. Figure 6 shows the pooled analysis of the animals exposed to 5 or 20 Gy (individual curves are reported in Supplementary Fig. 5). The results clearly show a significant difference between the low- and high-dose experiments. The fast component, very well visible at 5 Gy, essentially disappears at 20 Gy. This suggests a quick vascular injury at high doses that delays the washout process in the first half an hour after the irradiation.

## Discussion

The goal of the BARB project was to provide a demonstration of tumour treatment with RIB with online range verification by PET. The results in Fig. 5 demonstrate successful tumour control with RIB. We observed only minor toxicity, correlated to the residual activity measured in the spine (Extended Data Fig. 8). We conclude that image-guided particle therapy with RIB is feasible, safe and effective.

As previously observed in phantom experiments^44^, even using RIB for online beam imaging the activity peak can be shifted compared with the fall-off of the SOBP, depending on the beam momentum spread. In our study (Fig. 4a), the measured activity is shifted compared with the simulation in the in vivo experiment owing to anatomical changes (different horizontal/vertical position for planning/delivery), in addition to other uncertainties in the beam modelling, μCT calibration and imaging process. The anatomical changes after repositioning of the mouse from a horizontal to a vertical orientation (Extended Data Fig. 5) is particularly significant in light of ongoing efforts to implement particle therapy in an upright position^45^. Our findings reinforce the necessity of vertical CT planning and highlight the potential of online PET as a valuable tool for upright particle therapy.

Notwithstanding these uncertainties, our results show that the most distal position of the activity maximum from the Bragg peak can be used as a reliable indicator of the deepest location where a relevant amount (≥80%) of dose is deposited (Fig. 4b). The capability of RIB to predict the distal fall-off position around the 80% of the Bragg peak had been already demonstrated in phantoms^46^ and is now confirmed in vivo. Therefore, our results provide experimental support to the modelling predictions regarding the benefits of RIB in particle therapy, particularly for reducing target margins^19^.

The results on washout (Fig. 6) are intriguing. One of the main tenets of radiotherapy is that tumour control can be achieved only when all cancer stem cells are killed^47^. However, already 20 years ago, it was shown that microvascular endothelial apoptosis can contribute to tumour sterilization at high doses^48^. Later studies showed that the tumour damage at high doses induces vascular damage^49^ and can be mediated by an ischaemic–reperfusion mechanism^50^. This idea has already been translated into clinical practice with single-dose radiotherapy (SDRT)^51^, which uses single fractions of 24 Gy rather than fractionation in small malignancies, achieving excellent clinical results^52^. However, this concept is controversial. In fact, according to the classical linear-quadratic model, single fractions are much more effective than fractionated doses, and therefore the benefits of SDRT may simply be attributed to the high biological effectiveness of single doses^53,54^. The available experimental data are not conclusive^55^. Dynamic contrast-enhanced magnetic resonance imaging in rats shows increased permeability after high doses of X-rays or C ions^56^, but those studies look at the effects weeks after irradiation, whereas our results cover the initial 30 min after exposure. This time frame is crucial, as other studies reported ischaemic stress following SDRT within a few minutes^50^ or hours^57^. Biological washout provides a direct measurement of the vascular perfusion in the tumour, and therefore we believe that this technique can clarify the vascular engagement after radiotherapy. Our results are consistent with an ischaemic stress occurring very early after high doses. Although reperfusion was not observed within the measured time interval, we cannot rule out the possibility that it may occur at a later time.

## Outlook

What are the next preclinical steps in RIB research? We will test short-lived isotopes such as ^10^C or ^15^O, which are expected to provide stronger signal and faster feedback, for increased temporal resolution. For ^10^C, it will be necessary to use the new Super-FRS^58^ at FAIR, which will be able to provide much higher intensities of secondary beams.

For the washout studies, future experiments should investigate a wider range of doses and extended post-irradiation timepoints, alongside tumour histology, to better assess vascular changes after irradiation.

Can these successful results lead to a clinical translation of RIB? The MEDICIS-Promed^59,60^ project at CERN proposed an isotope separation on-line (ISOL) production of a ^11^C beam that can then be injected directly into medical synchrotrons currently used for ^12^C-ion therapy. Moving the isotope production to the low-energy injecting area can indeed be a feasible solution where RIB can be used at least for an initial test of the range before a full treatment course. The Open-PET scanner^61,62^ developed at National Institutes for Quantum Science and Technology (QST) in Japan or the INSIDE in-beam PET^10,63^ installed at CNAO in Italy can be excellent detectors for clinical applications of RIB. Finally, washout may also hold important prognostic value in the clinic, as it is expected to correlate with the tumour’s vascular state and potentially with the level of hypoxia^43,64^, a well-known negative prognostic factor in therapy^65^. Our preclinical results show the feasibility of RIB radiotherapy and support these ongoing efforts for clinical translation.

## Methods

### RIB production

The ^11^C beam was produced via in-flight separation. A 300 MeV n^−1^ ^12^C primary beam from the SIS18 synchrotron impinges on a 8.045 g cm^−2^ thick beryllium target at the FRS^36^ and undergoes peripheral nuclear reactions, so that one or more nucleons are stripped off, leading to a variety of lighter isotopes from carbon and other elements from boron down to hydrogen. Via a combined magnetic rigidity analysis and energy loss, which is induced in a so-called wedge-shaped degrader that is located at the central focal plane of the FRS, an isotopic clean ^11^C beam is achieved. Event-by-event particle identification using *Bρ*–Δ*E*–TOF, where *Bρ* is magnetic rigidity, Δ*E* is energy loss and TOF is time-of-flight, was measured in a previous, preparatory experiment. The purity of the beam was about 98% (Supplementary Fig. 6). This beam was used at the FRS for a variety of basic nuclear and atomic physics studies (such as reaction cross-sections of the ^11^C ions, their range and range straggling, basic PET studies and so on)^24,25,66^ in preparation of the present experiment. Via the connecting beamline to the target hall^26^, the isotopic clean beam is transported to Cave M, where the present irradiation was accomplished. The FRS and its three branches are shown in Extended Data Fig. 1, and exact parameters of the beam used in the present experiments are reported in Extended Data Table 1.

### Dosimetry

The ^11^C beam from the FRS reached the experimental room with an intensity of 2.5 × 10^6^ particles per spill. To minimize irradiation and allow for extended PET image acquisition, a beam cycle of 0.2 s ON and 3 s OFF was used. Once in the experimental room, the beam was monitored with large parallel plate ionization chambers^67^. The beam was characterized in terms of beam spot size and one-dimensional and 3D depth dose distributions in water by means of a PTW PEAKFINDER^T^ system (PTW Freiburg) and an in-house water phantom^68^ equipped with an OCTAVIUS 1600 XDR (PTW Freiburg). The latter set-up allows the acquisition of 2D dose distributions at different water depths, which can be processed to generate 3D dose map distributions.

A range modulator (Extended Data Fig. 3a) was used to generate a 1.2-cm SOBP in water. This modulator was 3D-printed on a 3D Systems ProJet MJP 2500 Plus using VisiJet M2S-HT250 as the printing material and VisiJet M2 SUP as the support material. The printing material has a water-equivalent density of 1.162 g cm^−3^ and a physical density of 1.1819 g cm^−3^.

The measured pristine and SOBP curves are shown in Extended Data Fig. 2. The Bragg peak position in water for the pristine depth dose distribution was measured at 80.5 mm. By comparison with Monte Carlo simulations, it was then possible to estimate beam parameters such as the beam energy and momentum spread. All measured and estimated beam parameters, also used for the Monte Carlo simulations, are reported in Extended Data Table 1.

As the ^11^C beam spot size was much larger than the standard clinical beam and the size of the target volume was comparably small, it was not possible to use standard active scanning techniques to deliver the desired dose to the CTV. A system of modulator, degraders, collimator and compensator was then used to passively modulate and optimize the beam for animal irradiation.

A schematic of the complete set-up for mice irradiation is shown in Fig. 2a, and a more detailed description of the experimental set-up and beam characterization can be found in ‘Dosimetry’ in the Supplementary Information. At first, to achieve the desired penetration depth in the mouse neck (~5.6 mm), the energy of the beam at the mouse position had to be reduced. This was achieved by introducing defined thicknesses of material in the beamline, in particular, 28.4-mm-thick aluminium plates (60.1 mm water equivalent path length), and a range shifter equipped with remotely controlled polyethylene plates. This latter component allowed fine adjustments of the Bragg peak position and a dose delivery correction in case a range correction would have appeared necessary during the treatment. The distal edge of the SOBP was shaped to the distal contour of the generalized CTV using a specially designed compensator (Extended Data Fig. 3b), which was placed on the neck of the mice and secured on the bed. These compensators, produced using the same 3D printer as the range modulator, also served to immobilize and position mice.

To laterally define the irradiation area, in addition to the mouse neck compensator, a system of brass collimators with a 15 × 12 mm elliptical aperture was placed right before the PET scanner (Fig. 2a and Supplementary Fig. 7). This system had also the function of shielding the detector and limiting the noise signal in the scanner by blocking most of the ions that would not contribute to the dose on the target. Absolute dosimetry at the tumour site was performed using a small-volume pinpoint ionization chamber (PTW TM31023) placed within a custom-designed dosimetry holder (see ‘Dosimetry’ in the Supplementary Information; Supplementary Figs. 13 and 14). This set-up ensured that the detector’s sensitive volume corresponded to the tumour depth. The absolute dose measured by the chamber was then used to determine a set-up-specific monitor unit calibration, allowing the scaling of treatment plans to the prescribed dose. The chamber readings were recorded using a PTW UNIDOS electrometer, applying the standard correction factors (*k*_Q_ and *k*_TP_) commonly used in particle therapy (see ‘Dosimetry’ in the Supplementary Information for further details).

### Animal model

All experiments were performed using 11–12-week-old female C3H/He mice (*Mus musculus*) purchased from Janvier Labs, according to German Federal Law under the approval of the Hessen Animal Ethics Committee (Project License DA17/2003, Regierungspräsidium Darmstadt). Mice were divided into five groups: 20 Gy ^11^C irradiation (17 animals, followed up for 6 months after the irradiation); 20 Gy and 5 Gy ^11^C irradiation followed by additional 30 min of PET signal acquisition (8 and 7 animals, respectively, followed up for 4 weeks after the irradiation); tumour-bearing sham-irradiated controls (27 animals, followed up for 4 weeks after the irradiation day); and tumou-free controls (8 animals, followed up for 6 months after the irradiation day). Mice were housed at GSI in a conventional animal facility (non-specific-pathogen-free) at 22 °C and 55% humidity, 12-h light–dark cycle, with unrestricted access to water and a standard diet (Ssniff). Fourteen days before irradiation, 10^6^ mouse Dunn osteosarcoma LM8 cells (originating from C3H/He mice, purchased from Riken BioResource Center) were injected in 20 µl of phosphate-buffered saline buffer solution subcutaneously in the neck area of the mouse, above the cervical area of the spine. To maintain the consistency of injections, during the procedure animals were anaesthetized with 2% isoflurane, which was inhaled via a face mask. After 2 weeks, most tumours were palpable and measurable, at least with μCT.

### CT imaging

#### Horizontal imaging—μCT

To collect the data on the tumour growth, we performed μCT measurements with a VivaCT 80 scanner (SCANCO Medical AG). To ensure the reproducible positioning of the mice as during the ^11^C irradiation, we used a custom-made bed imitating the geometry of the SIRMIO bed. In addition, animals were immobilized using the compensator so that the scans could be utilized for the later Monte Carlo calculations. During the scan, animals were anaesthetized with isoflurane (3% for the induction, 1.5–2% for maintenance during the scan). Neck regions of 31 mm were scanned for approximately 5 min at a tube voltage and current of 45 kVp and 177 μA, respectively, adding a 0.1-mm aluminium filter, acquiring 250 projections per 180° with 45 ms integration time. The resulting images had a voxel size of 97.1 μm. After the scan, animals were allowed to recover before being transferred to the original cage. The scans performed at 14 days after the tumour cell injection into the animals used to establish the tumour model were used to contour the generalized CTV for the treatment planning. The contouring of the visible tumour mass was done manually with the 3D Slicer 5.0.3 software^69^ for every animal; then the individual GTV contours were added up. We expected that the majority of the tumours in the ^11^C groups would grow in similar locations and not differ in size from those of the test group. Nevertheless, to increase robustness towards the biological variation, we smoothened the resulting CTV contour and made it symmetrical with respect to the spine (Fig. 1c). The animals selected for ^11^C irradiation were scanned one day before the irradiation (13 days after the tumour cell injection).

#### Vertical imaging—SARRP

To resolve remaining discrepancies between simulations and measurements (Fig. 4a), we checked for anatomical changes arising from repositioning the mice from the horizontal μCT bed, used for imaging, onto the vertical SIRMIO bed used during the ^11^C irradiation (Fig. 2). We utilized the CBCT of the Cave M SARRP (XStrahl) to acquire vertical CT scans of mice by positioning them onto a vertical holder replicating the geometry and fixation procedure (including the compensator) of the SIRMIO bed.

During these tests, animals were anaesthetized with isoflurane (3% for the induction, 1.5% for maintenance) and, to further imitate the conditions of the main experiment, they were left in the vertical position for several additional minutes before the scans were taken. We acquired 250 projections per 180° at a tube voltage and current of 70 kVp and 1 mA, respectively, adding a 0.1-mm copper filter. The resulting images had a voxel size of 275 μm. After the scan, animals were allowed to recover before being transferred to the original cage.

We observed some consistent variations in the spine curvature (<1.5 mm in the neck area) between the μCT and the CBCT scans. This anatomical change reconciles the measurements with the Monte Carlo simulations (Fig. 4b).

### Tumour vascularization

To assess the tumour vascularization, animals were euthanized and perfused ex vivo with Vascupaint contrast agent (yellow colloidal bismuth suspension, MediLumine). After allowing the compound to polymerize for 24 h, tumours were extracted and scanned at a high resolution (10 μm) with the following scanner settings: tube voltage and current of 55 kVp and 145 μA, respectively, 0.5-mm aluminium filter, 1,500 projections per 180° with 600 ms integration time. The 3D reconstruction of the tumour vasculature was done using the scanner’s built-in software ‘Bone morphology’ function following the approach of another study with a contrast agent^70^.

### Online PET

A spherical, high-resolution PET scanner developed at the Ludwig-Maximilian-University in the framework of the SIRMIO project was used to measure the RIB implantation in-beam during irradiation. The SIRMIO PET scanner features 56 three-layer depth-of-interaction (DOI) detectors arranged in a spherical shape with an inner diameter of 72 mm (ref. ^28^). Each DOI PET detector consists of a LYSO scintillator block with a pixel size of 0.9 mm readout by an 8 × 8 silicon photomultiplier (SiPM) array. A charge division circuit and a custom-made amplifier circuit board developed at the National Institutes for Quantum Science and Technology (Chiba, Japan) are used to reduce the 64 signals from the SiPM array to 4 signals. The data are then acquired by a customized DAQ software using two R5560 digitizers (CAEN, Italy). To enable image-guided irradiation for the BARB project, the data acquisition and reconstruction software were tailored to stream out and reconstruct the list mode data with user-defined time intervals, set in this experiment to cycles of 60 s. This feature enables visualizing the reconstructed stopping position of the beam online during the irradiation, along with the monitoring of the irradiation build up and decay through a graphical user interface. For this specific online application, the image reconstruction was based on an in-house developed ordered subset expectation maximization algorithm, with a reduced number of iterations and limited size of the field of view for the sake of computational speed during the experiment. The 3D activity maps and washout analyses used for the reported results were based on a more time-consuming 3D maximum likelihood expectation maximization with relevant corrections for sensitivity and random coincidences. Attenuation corrections were not included as they have a negligible influence on the shape of the activity distribution for the considered small size of the irradiated target.

### Co-registration

For accurate co-registration of the imaged activity with the pretreatment μCT, before the experiment a specially designed mouse bed equipped with an insert for a ^22^Na point source was positioned in the PET scanner. Multiple point-source measurements were performed at well-controlled positions using precision linear stages to move the SIRMIO bed with the source at different locations in the field of view of the SIRMIO PET scanner. By knowing both the physical location of the point source in the bed and its reconstructed position in the PET image, the mouse bed—and consequently the mouse position during treatment through the reproducible positioning of the mouse compensator on the bed—could be accurately aligned within the PET field of view, thus enabling an accurate overlay of the reconstructed activity images with the treatment planning anatomy.

### Monte Carlo

An extensive FLUKA Monte Carlo^71^ simulation study was conducted to support both the experiment design and its data analysis. Simulations were performed using the HADRONTherapy DEFAULT card in FLUKA (v2021.2.3) with the flair GUI (v2.3-0). The mean water ionization potential was set to 78 eV (ref. ^72^). We activated the COALESCEnce card for light fragment spectra and residual nuclei, and the IONSPLIT card for deuteron splitting at low-energy interactions. For simulation in the SOBP configuration, a user-defined USERROUTINE was implemented to read the 2DRM geometry file^73^. The number of primary particles in the simulations was chosen to ensure that statistical uncertainties due to Monte Carlo fluctuations remained below 1%.

The simulations assisted in designing beamline components, including the 2D range modulator, collimator and mouse compensator, and in developing shielding strategies to protect the SIRMIO PET scanner from radiation damage. As in our previous BARB dosimetry study, FLUKA simulations were also used to verify dosimetry measurements and support beam model characterization^66^ (see ‘Dosimetry’ in the Supplementary Information).

Expected dose and positron annihilation maps inside the body were simulated by importing the mice scans with their original resolution in a voxel FLUKA geometry. For the purpose of accurate beam model and transport, the full experimental set-up was implemented into the FLUKA geometry, and a set-up-specific CT number to stopping power calibration, including the mouse bed and compensator, was implemented in FLUKA.

To reproduce the detector response and imaging process, the annihilation maps simulated with FLUKA were then imported in a Geant4-dedicated simulation set-up that includes a detailed model of the SIRMIO PET scanner^26^. To start propagating the annihilation photons from the FLUKA simulated annihilation maps, positrons with no kinetic energy were simulated in Geant4 to enforce annihilation at the same position as the input map. The resulting annihilation photon pairs were then transported in air through the detector set-up, accounting for the geometrical detector response, but omitting attenuation in the target (as it has a negligible effect on the shape of the reconstructed activity and is also not applied as correction in the image reconstruction). The Geant4 simulation output is a list of hits in the detector crystals, which is then postprocessed to resemble the experimental data.

The final simulated PET image was obtained using the same reconstruction method applied to the real measurements. The correction for sensitivity used in the reconstruction is based on the same Monte Carlo simulation model, which was validated at a few positions in the relevant central part of the field of view where the maximum of activity is expected. Small remaining inconsistencies between the theoretical and real sensitivity at the edge of the field of view could affect the reconstruction of the simulated and measured data differently, introducing small mismatches of different magnitude in the entrance or tail region, which are visible in the profiles in Fig. 4. Moreover, the simulated PET images are based only on the simulated annihilation distributions and do not include the prompt radiation background generated by the RIB irradiation, nor the very minor amount of intrinsic radioactivity of the LYSO crystals used^28^. Furthermore, the simulation does not include the biological washout model, which can slightly broaden the measured activity in the animal. However, the radiation background was largely suppressed in the measured data by using very narrow coincidence energy windows, exploiting the large signal-to-noise ratio of RIB irradiation. Moreover, the washout contribution can be considered negligible in the first few minutes of irradiation, in which the output of our online monitoring was used to decide on the adequate sparing of the spine.

### Mouse follow-up

#### Tumour growth

Starting 1 week after the injection, tumour dimensions were measured with a caliper twice per week for 28 days after the irradiation (Fig. 5a). Assuming the ellipsoid shape of the tumour, the volume was calculated as

2 $$V=\frac{4}{3}\uppi {abc},$$

where *a* and *b* are the measured length and width of the tumour, respectively, and *c* (depth) is assumed to be the average of *a* and *b*. For more accurate and reproducible estimates^74^, volumes were also measured using the μCT (Fig. 5b). After the irradiation, animals were scanned weekly for 4 weeks. As specified in the Gesellschaft für Versuchstierkunde (GV-SOLAS) guidelines and in the project ethical licence, the maximum permitted tumour size is 1.5 cm in diameter. When reached, the animals were euthanized according to the ethical protocol to not exceed the permitted burden. Animals remaining after the 28-day timepoint were scanned monthly afterwards until the euthanasia timepoint.

#### Toxicity assays

##### Skin toxicity scoring

The toxicity of the skin in the irradiated area was scored using a simplified grading system of the GV-SOLAS guidelines, divided by five grades (0: no effect; 1: redness; 2: dry skin and desquamation; 3: closed, healing wound; 4: open wound, not healing; 5: necrosis). Grade 5 was never observed during the experiment, and the termination criteria were reached when the animals showed grade 4 and did not heal after the application of a topical treatment (Bepanthen, Bayer). The treatment healed successfully the animals with grade 3 after irradiation.

##### Grip test

The grip test was performed to measure the strength of the animals’ forelimbs after irradiation. Animals were acclimatized to refined handling techniques to reduce stress and optimize the data collection. We used the Grip Strength Meter-47200 (Ugo Basile) equipped with a T-bar and built-in data collection agent (DCA) software. The animals were lifted by the tail and suspended over the bar, then lowered to reach a horizontal position and gently pulled back until the grasp was released. Upon release, the peak force (in newton) was recorded. To get consistent data and avoid habituation to the task, the first three measurements in which the animal successfully grabbed the bar with both forelimbs were recorded and averaged. The procedure is illustrated in Extended Data Fig. 8.

##### Kyphosis scoring

To score the overall appearance and health status of the animals, videos were recorded in a house-made set-up consisting of a starting box, a transparent-walled corridor and a loop structure at the end, where the animals could enter and go back to the corridor. The animals were observed for spontaneous walking, grooming behaviour and posture during stationary and movement phases. To evaluate the kyphosis, we used the scoring system from ref. ^75^ where in grade 0 there is no persistent kyphosis and the mouse can always straighten the spine; in grade 1, mild kyphosis is exhibited during stationary phase, but the spine is straightened during locomotion; in grade 2, persistent mild kyphosis is observed even during movement, and the spine cannot be straightened completely; and in grade 3, the kyphosis is always maintained and well pronounced.

### Statistics and reproducibility

For the animal irradiation experiments, we chose sample sizes for an expected effect size (Cohen’s *d*) of *d* = 1. Sample sizes were determined online using G*Power software version 3.1.9.7. Animals were randomly allocated into experimental groups. During the animal follow-up (tumour size measurements and grip strength measurements), the investigators were blinded to group allocation. Where applicable, data distribution was assumed to be normal, but this was not formally tested. The details of the individual statistical tests used for data analysis are specified in detail in the respective figure captions. Washout data from one animal in the 20 Gy group (mouse ID 98; Supplementary Fig. 5) was excluded from the analysis due to being identified as statistical outlier, as it exceeded 1.5× the interquartile range from the group medians.

### Reporting summary

Further information on research design is available in the Nature Portfolio Reporting Summary linked to this article.

## Online content

Any methods, additional references, Nature Portfolio reporting summaries, source data, extended data, supplementary information, acknowledgements, peer review information; details of author contributions and competing interests; and statements of data and code availability are available at 10.1038/s41567-025-02993-8.

## Supplementary information

Supplementary Information Supplementary figures and dosimetry.

Reporting Summary

Supplementary Video 1 ^11^C activity in irradiated mouse. Time-resolved build-up of the PET signal monitored online during the mouse irradiation. The image is overlaid on the mouse μCT in sagittal (left) and transversal (right) views (see section co-registration in the Methods). Time and dose are also indicated.

Supplementary Video 2 Vascular structure of the tumour. Three-dimensional rendering of the vascular structure of the LM8 osteosarcoma tumour as visualized in μCT after staining with Vascupaint. Quantitative values are shown in the table.

Source Data for Supplementary Fig. 3

## Figures and Tables

**Extended Data Table 1:**
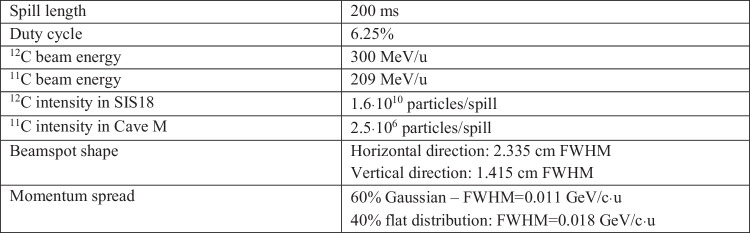
Summary of the beam parameters used in the experiments

## Data Availability

Raw data for all plots are publicly available via Figshare at 10.6084/m9.figshare.27102097 (ref. ^76^). Raw data for the PET images and DICOM of the μCT are available on request to the corresponding author.
