# Supplementary material for: Image-guided treatment of mouse tumours with radioactive ion beams
Source: Nat Phys. Author manuscript; Available in PMC 2025 Oct 16. (PMC12518140; doi:10.1038/s41567-025-02993-8)
Supplement: Supplementary Materials [file EMS207960-supplement-Supplementary_Materials.zip › 41567_2025_2993_MOESM2_ESM.pdf]

nature portfolio

Corresponding author(s): Durante, Marco

Last updated by author(s): 17/06/2025

Reporting Summary

Nature Portfolio wishes to improve the reproducibility of the work that we publish. This form provides structure for consistency and transparency in reporting. For further information on Nature Portfolio policies, see our Editorial Policies and the Editorial Policy Checklist.

Statistics

For all statistical analyses, confirm that the following items are present in the figure legend, table legend, main text, or Methods section.

n/a	Confirmed	
[ ]	[x]	The exact sample size (*n*) for each experimental group/condition, given as a discrete number and unit of measurement
[ ]	[x]	A statement on whether measurements were taken from distinct samples or whether the same sample was measured repeatedly
[ ]	[x]	The statistical test(s) used AND whether they are one- or two-sided<br>*Only common tests should be described solely by name; describe more complex techniques in the Methods section.*
[ ]	[x]	A description of all covariates tested
[ ]	[x]	A description of any assumptions or corrections, such as tests of normality and adjustment for multiple comparisons
[ ]	[x]	A full description of the statistical parameters including central tendency (e.g. means) or other basic estimates (e.g. regression coefficient) AND variation (e.g. standard deviation) or associated estimates of uncertainty (e.g. confidence intervals)
[ ]	[x]	For null hypothesis testing, the test statistic (e.g. *F*, *t*, *r*) with confidence intervals, effect sizes, degrees of freedom and *P* value noted<br>*Give P values as exact values whenever suitable.*
[x]	[ ]	For Bayesian analysis, information on the choice of priors and Markov chain Monte Carlo settings
[x]	[ ]	For hierarchical and complex designs, identification of the appropriate level for tests and full reporting of outcomes
[ ]	[x]	Estimates of effect sizes (e.g. Cohen's *d*, Pearson's *r*), indicating how they were calculated

Our web collection on statistics for biologists contains articles on many of the points above.

Software and code

Policy information about availability of computer code

Data collection	Dosimetry data: PTW PEAKFINDERTM system (PTW Freiburg, Germany), OCTAVIUS 1600 XDR (PTW Freiburg, Germany)<br>PET data: customized DAQ software using two R5560 digitizers (CAEN, Italy)<br>CT images: VivaCT 80 scanner (SCANCO Medical AG, Switzerland) and Small Animal Radiation Research Platform (SARRP, XStrahl, USA)
Data analysis	Python 3.10<br>HADRONTherapy DEFAULT card in FLUKA (v2021.2.3) with the flair GUI (v2.3-0)<br>GraphPad Prism 10.5.0 (774)<br>R v.4.2.1 ggplot packge<br>3D Slicer 5.3.0<br>Microsoft Office Professional Plus Excel 2016

For manuscripts utilizing custom algorithms or software that are central to the research but not yet described in published literature, software must be made available to editors and reviewers. We strongly encourage code deposition in a community repository (e.g. GitHub). See the Nature Portfolio guidelines for submitting code & software for further information.

Data

Policy information about availability of data

All manuscripts must include a data availability statement. This statement should provide the following information, where applicable:

- Accession codes, unique identifiers, or web links for publicly available datasets
- A description of any restrictions on data availability
- For clinical datasets or third party data, please ensure that the statement adheres to our policy

All data supporting the findings are present in the main text and /or supplemetrary materials. The raw data for all the plots are available in Figshare repository under the following link: https://doi.org/10.6084/m9.figshare.27102097 The raw data for the images are available on request.

Research involving human participants, their data, or biological material

Policy information about studies with human participants or human data. See also policy information about sex, gender (identity/presentation), and sexual orientation and race, ethnicity and racism.

Reporting on sex and gender	The study did not involve human participants, their data, or biological material
Reporting on race, ethnicity, or other socially relevant groupings	The study did not involve human participants, their data, or biological material
Population characteristics	The study did not involve human participants, their data, or biological material
Recruitment	The study did not involve human participants, their data, or biological material
Ethics oversight	The study did not involve human participants, their data, or biological material

Note that full information on the approval of the study protocol must also be provided in the manuscript.

Field-specific reporting

Please select the one below that is the best fit for your research. If you are not sure, read the appropriate sections before making your selection.

☒ Life sciences ☐ Behavioural & social sciences ☐ Ecological, evolutionary & environmental sciences

For a reference copy of the document with all sections, see nature.com/documents/nr-reporting-summary-flat.pdf

Life sciences study design

All studies must disclose on these points even when the disclosure is negative.

Sample size	For the animal irradiation experiments, we chose sample sizes for an expected effect size (Cohen's d) of d=1. Sample sizes were determined online using G*Power software version 3.1.9.7.
Data exclusions	Washout data from one animal in the 20 Gy group (mous ID 98, see Supplementary Figure 4) was excluded from the analysis presented in Figure 6 due to being identified as statistical outlier, as it exceeded 1.5x the interquartile range from the group medians.
Replication	*Describe the measures taken to verify the reproducibility of the experimental findings. If all attempts at replication were successful, confirm this OR if there are any findings that were not replicated or cannot be reproduced, note this and describe why.*
Randomization	Animals were randomly allocated into experimental groups.
Blinding	During the animal follow up (tumor size measurements, grip strength measurements) the investigators were blinded to group allocation.

Reporting for specific materials, systems and methods

We require information from authors about some types of materials, experimental systems and methods used in many studies. Here, indicate whether each material, system or method listed is relevant to your study. If you are not sure if a list item applies to your research, read the appropriate section before selecting a response.

Materials & experimental systems

n/a	Involved in the study	
☒	☐	Antibodies
☐	☒	Eukaryotic cell lines
☒	☐	Palaeontology and archaeology
☐	☒	Animals and other organisms
☒	☐	Clinical data
☒	☐	Dual use research of concern
☒	☐	Plants

Methods

n/a	Involved in the study	
☒	☐	ChIP-seq
☒	☐	Flow cytometry
☒	☐	MRI-based neuroimaging

Eukaryotic cell lines

Policy information about cell lines and Sex and Gender in Research

Cell line source(s)	Mouse Dunn osteosarcoma LM8 cells originating from male C3H/He mice
Authentication	Cells were commercially purchased from Riken BioResource Center, Japan where they were obtained as described in https://doi.org/10.1002/(SICI)1097-0215(19980504)76:3<418::AID-IJC21>3.0.CO;2-5<br>Cells were authenticated morphologically by microscopy in vitro.
Mycoplasma contamination	The cell lines are tested negative for mycoplasma contamination.
Commonly misidentified lines (See ICLAC register)	No misidentified cell line was used in the study.

Animals and other research organisms

Policy information about studies involving animals; ARRIVE guidelines recommended for reporting animal research, and Sex and Gender in Research

Laboratory animals	11-12-week-old female C3H/He mice (Mus musculus) purchased from Janvier Labs, France.
Wild animals	No wild animals were involved in the study.
Reporting on sex	The findings apply ony for one sex (female); no animal sex was considered in the study design.
Field-collected samples	The study did not involve samples collected in the field.
Ethics oversight	German Federal Law under the approval of the Hessen Animal Ethics Committee (Project License DA17/2003, Regierungspräsidium Darmstadt)

Note that full information on the approval of the study protocol must also be provided in the manuscript.

Plants

Seed stocks	No seed/plant material used in the study
Novel plant genotypes	No seed/plant material used in the study
Authentication	No seed/plant material used in the study
