# Supplementary material for: Image-guided treatment of mouse tumours with radioactive ion beams
Source: Nat Phys. Author manuscript; Available in PMC 2025 Oct 16. (PMC12518140; doi:10.1038/s41567-025-02993-8)
Supplement: Supplementary Materials [file EMS207960-supplement-Supplementary_Materials.zip › 41567_2025_2993_MOESM1_ESM.pdf]

nature physics

Supplementary information

https://doi.org/10.1038/s41567-025-02993-8

Image-guided treatment of mouse tumours with radioactive ion beams

In the format provided by the authors and unedited

Supplementary Figure 1. PET images of individual mice. Measured activity for all irradiated mice. In the background are shown the axial (left) and sagittal (right) μCT views of the corresponding mouse. All images show the last frame of the online reconstruction. For the individual time-dependent images monitored during the irradiation. For a time-dependent image monitored during the irradiation, see Supplementary video 1. The image reconstruction shown in these figures was based on an in-house developed ordered subset expectation maximization (*OSEM*) algorithm, as it was used during the experiment (see Methods for details).

Measurment time [s] = 210   Beam on [s] = 150   Dose [Gy] = 2.50

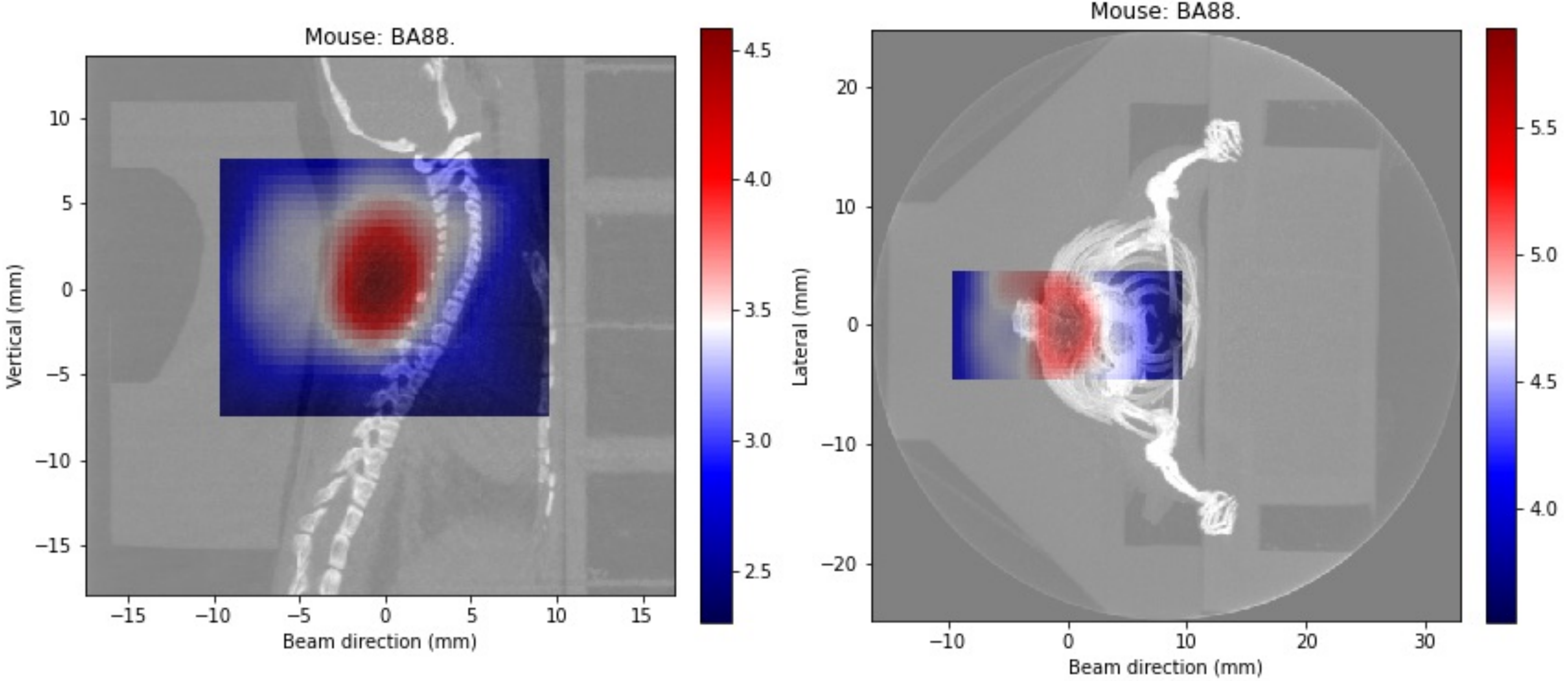

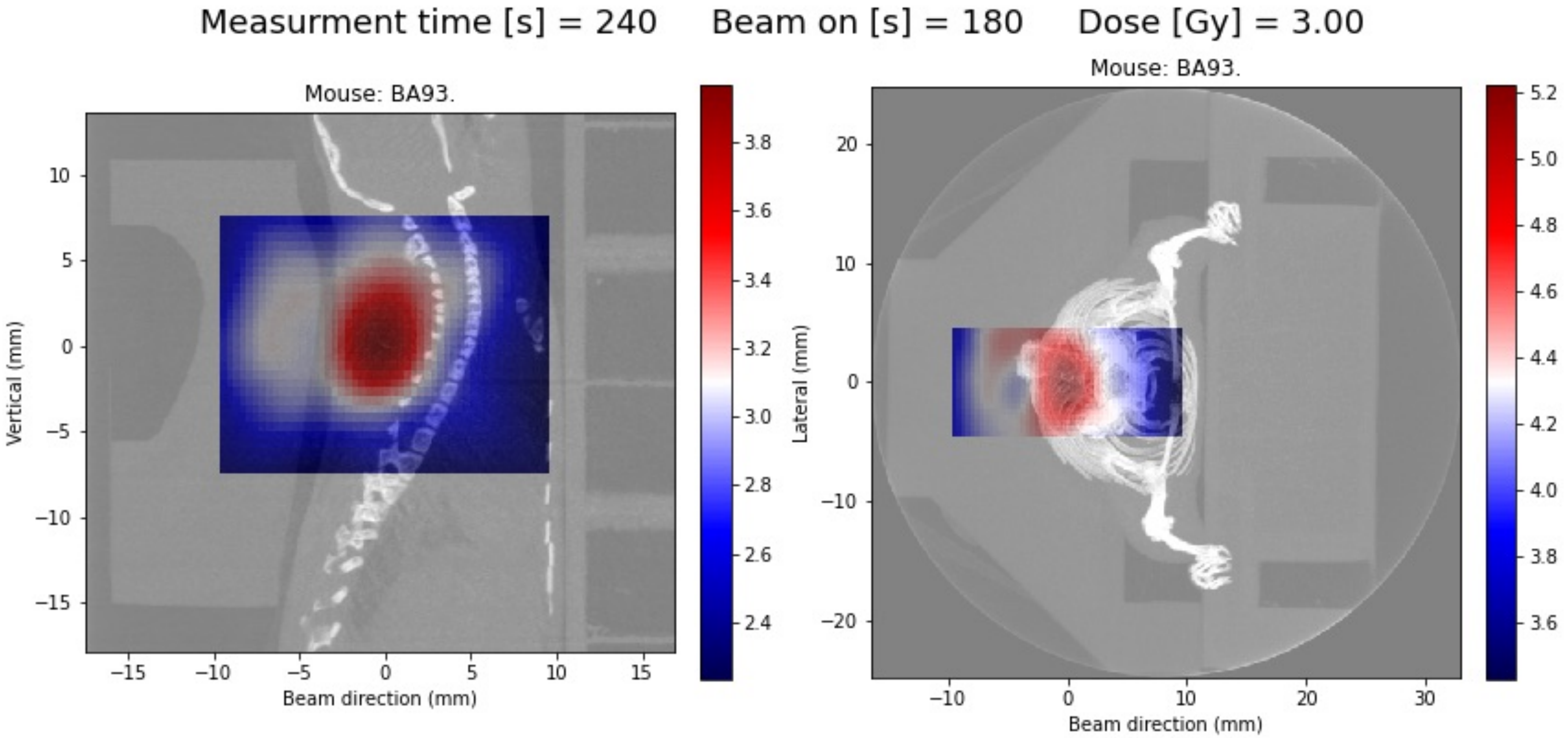
Measurment time [s] = 240 Beam on [s] = 180 Dose [Gy] = 3.00
Mouse: BA93.
Vertical (mm)
Beam direction (mm)
Mouse: BA93.
Lateral (mm)
Beam direction (mm)

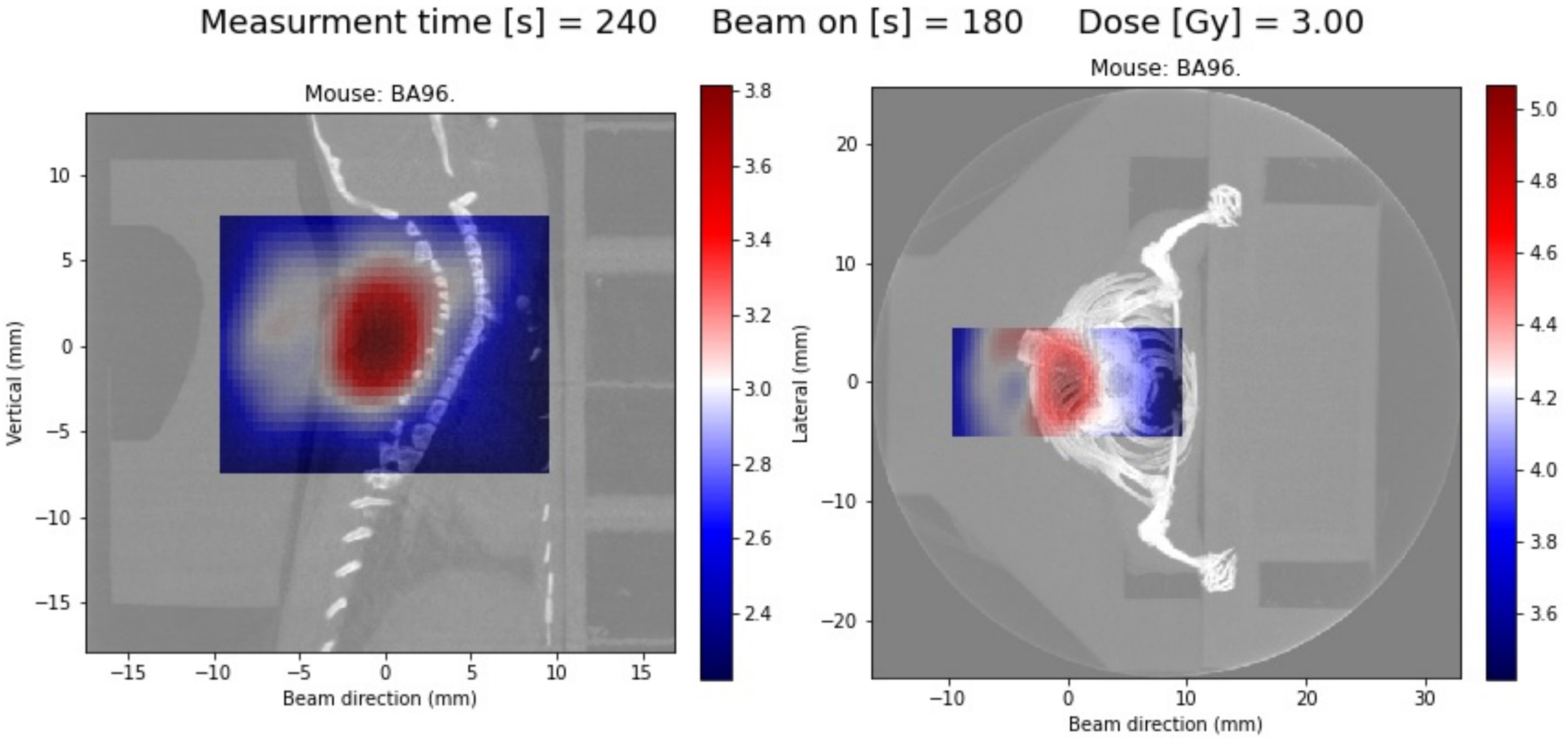
Measurment time [s] = 240 Beam on [s] = 180 Dose [Gy] = 3.00
Mouse: BA96.
Vertical (mm)
Beam direction (mm)
Mouse: BA96.
Lateral (mm)
Beam direction (mm)

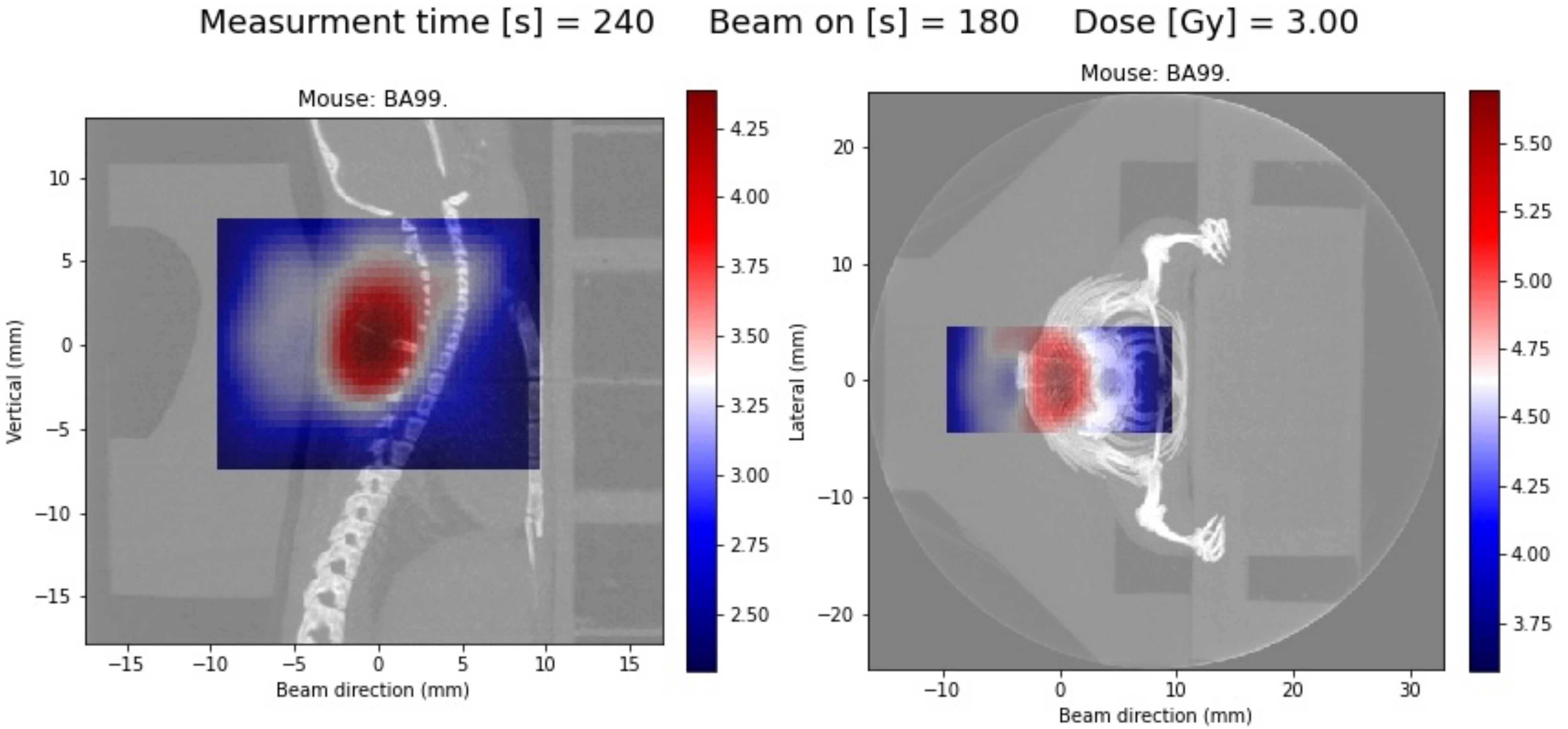
Measurment time [s] = 240 Beam on [s] = 180 Dose [Gy] = 3.00
Mouse: BA99.
Vertical (mm)
Beam direction (mm)
Mouse: BA99.
Lateral (mm)
Beam direction (mm)

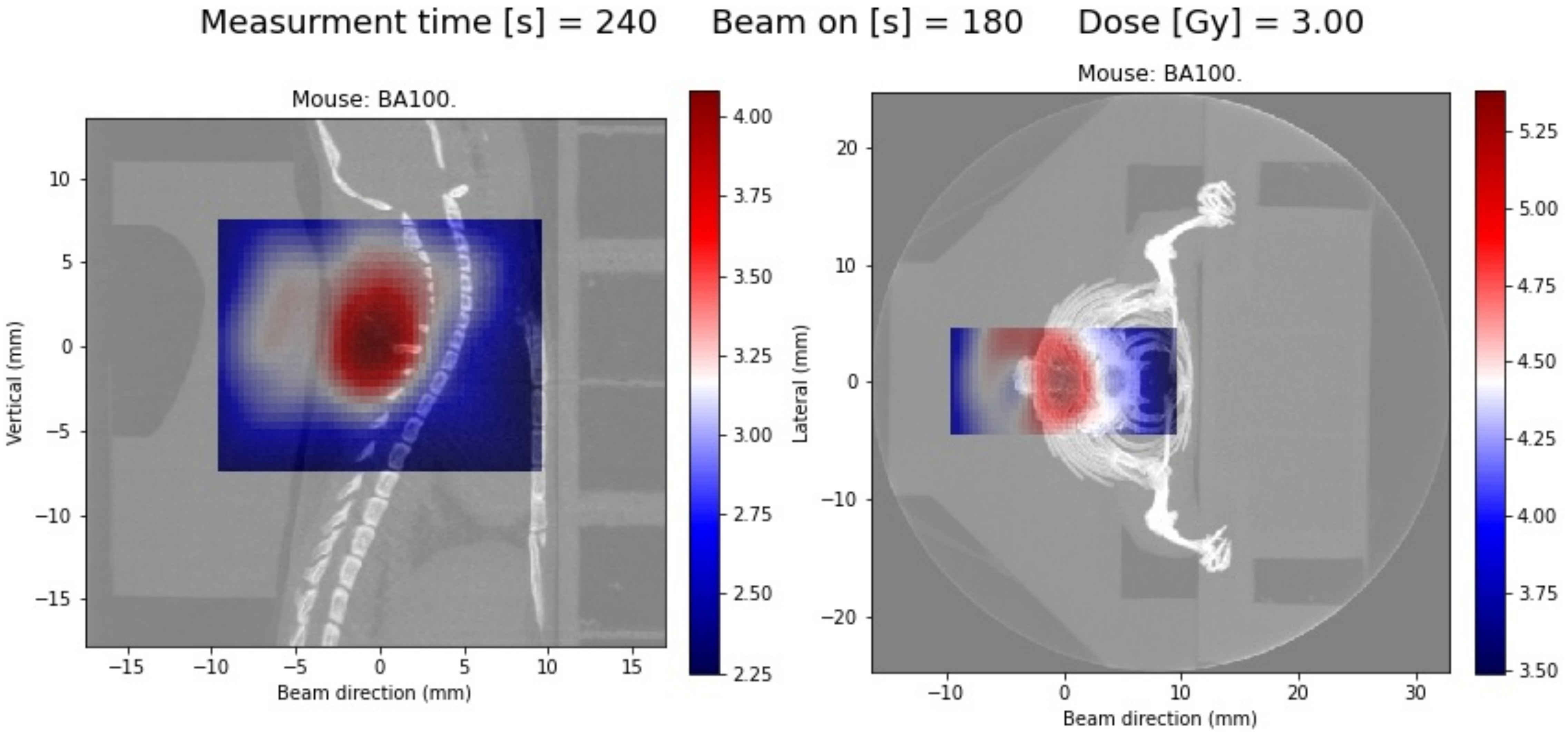

Measurment time [s] = 240 Beam on [s] = 180 Dose [Gy] = 3.00
Mouse: BA100.
Vertical (mm)
Beam direction (mm)
Mouse: BA100.
Lateral (mm)
Beam direction (mm)

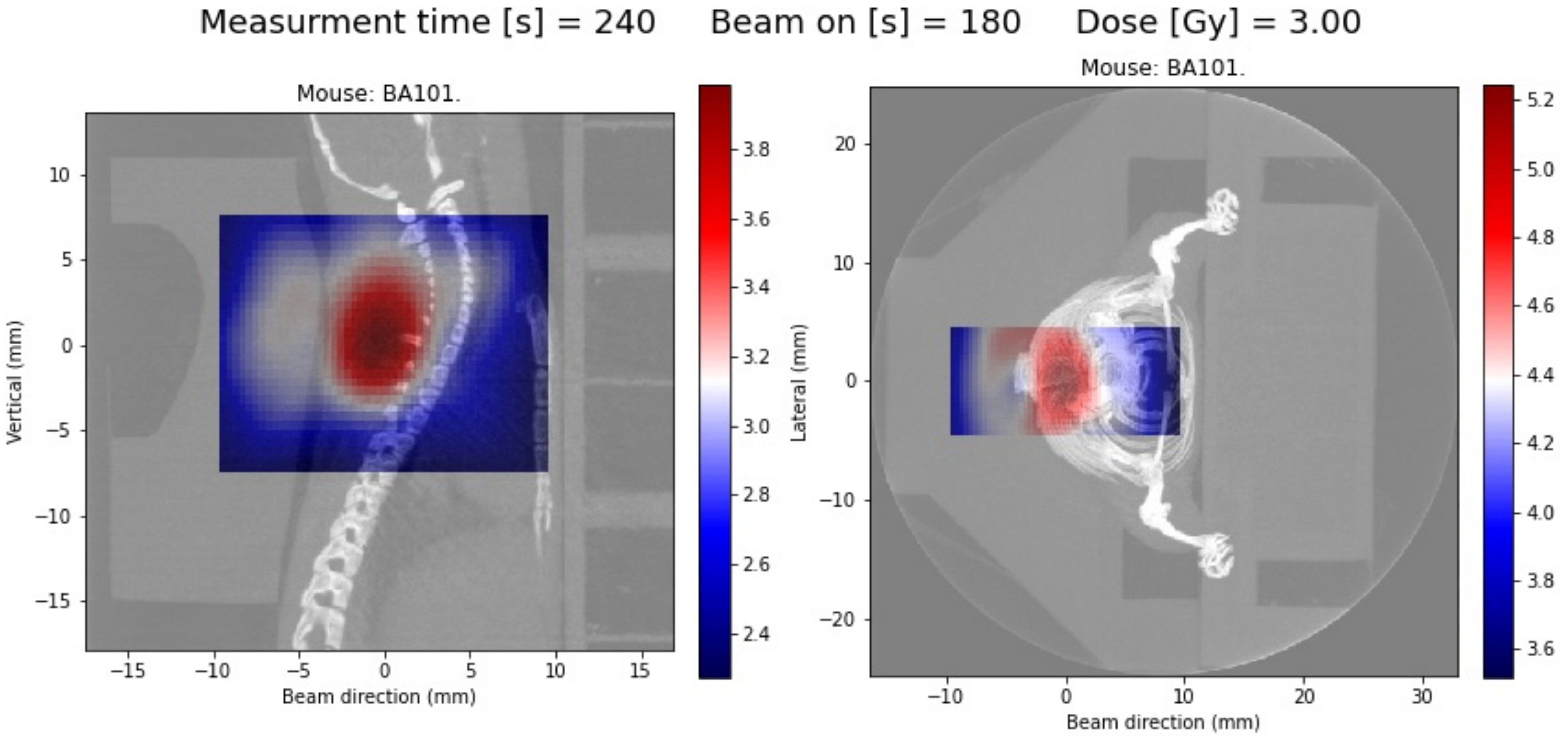
Measurment time [s] = 240 Beam on [s] = 180 Dose [Gy] = 3.00
Mouse: BA101.
Vertical (mm)
Beam direction (mm)
Mouse: BA101.
Lateral (mm)
Beam direction (mm)

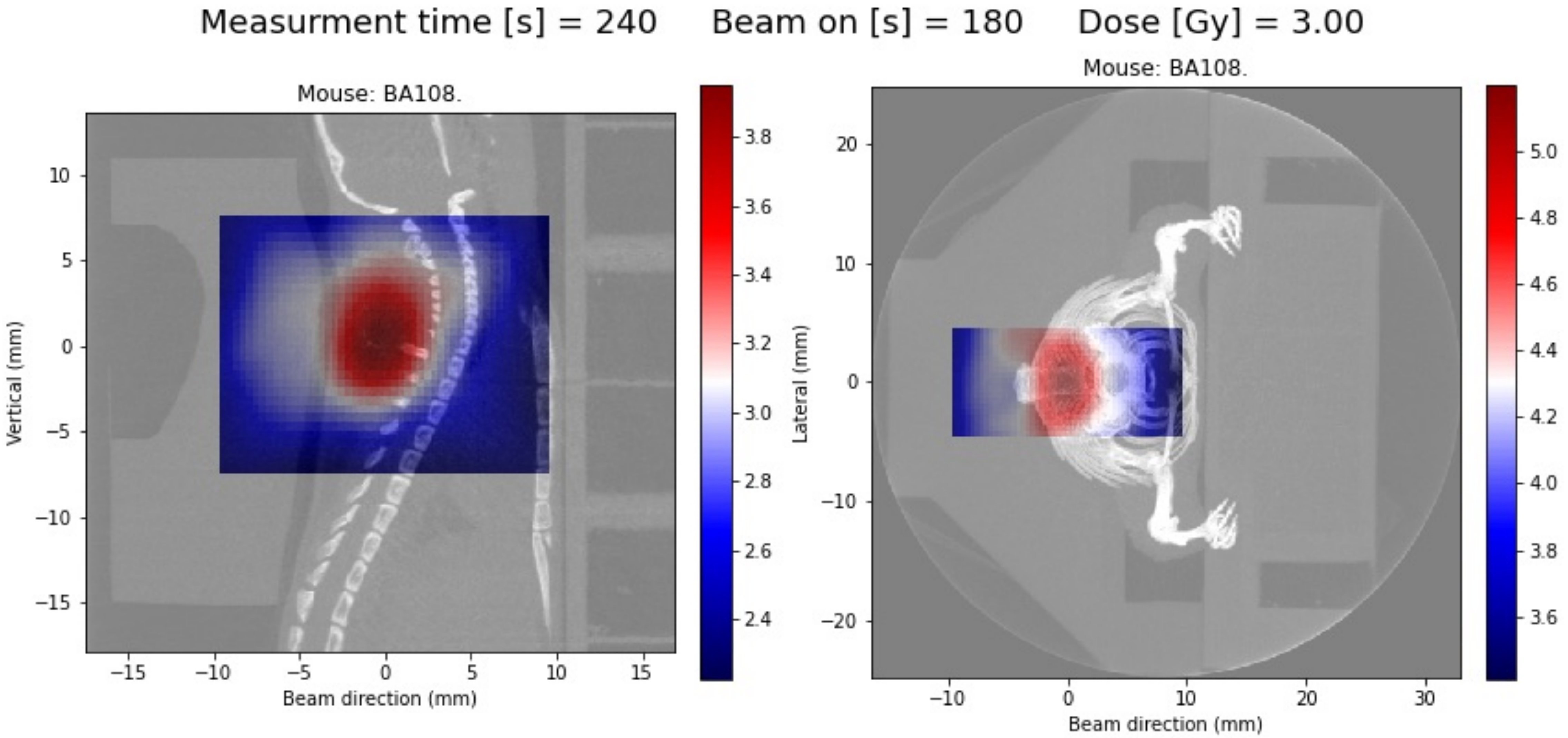
Measurment time [s] = 240 Beam on [s] = 180 Dose [Gy] = 3.00
Mouse: BA108.
Vertical (mm)
Beam direction (mm)
Mouse: BA108.
Lateral (mm)
Beam direction (mm)

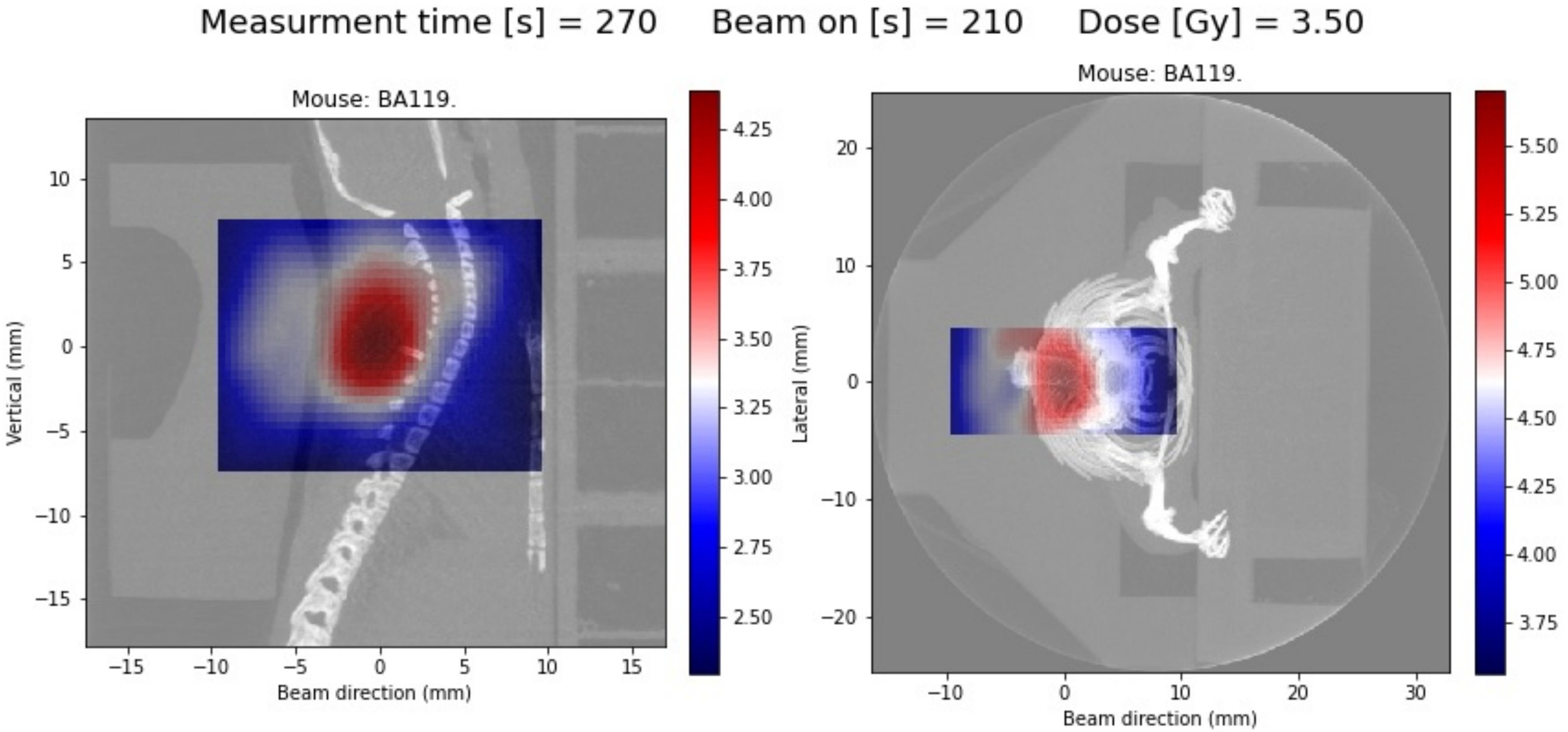
Measurment time [s] = 270 Beam on [s] = 210 Dose [Gy] = 3.50
Mouse: BA119.
Vertical (mm)
Beam direction (mm)
Mouse: BA119.
Lateral (mm)
Beam direction (mm)

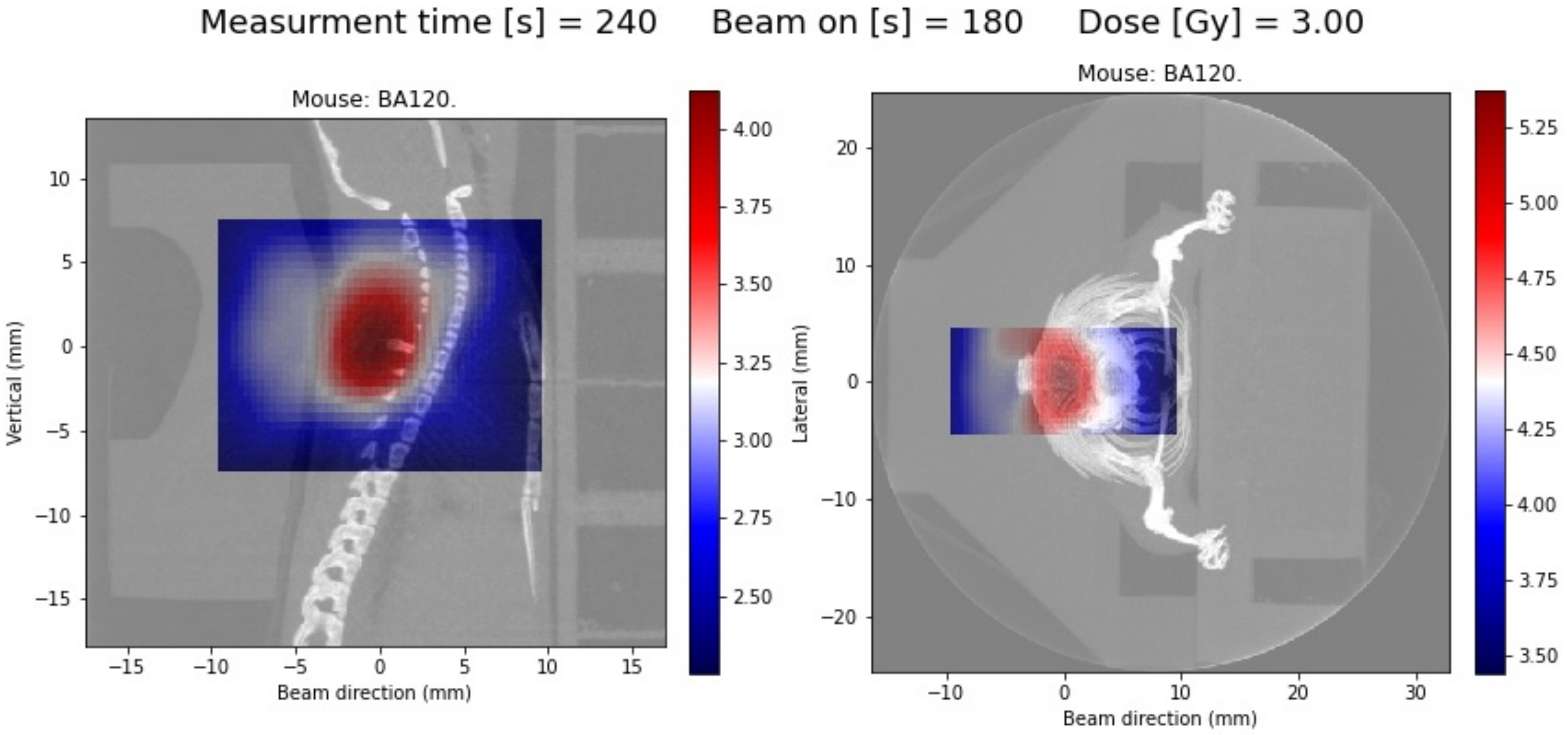

Measurment time [s] = 240 Beam on [s] = 180 Dose [Gy] = 3.00
Mouse: BA120.
Vertical (mm)
10
5
0
−5
−10
−15
−15
−10
−5
0
5
10
15
Beam direction (mm)
4.00
3.75
3.50
3.25
3.00
2.75
2.50
Mouse: BA120.
Lateral (mm)
20
10
0
−10
−20
−10
0
10
20
30
Beam direction (mm)
5.25
5.00
4.75
4.50
4.25
4.00
3.75
3.50

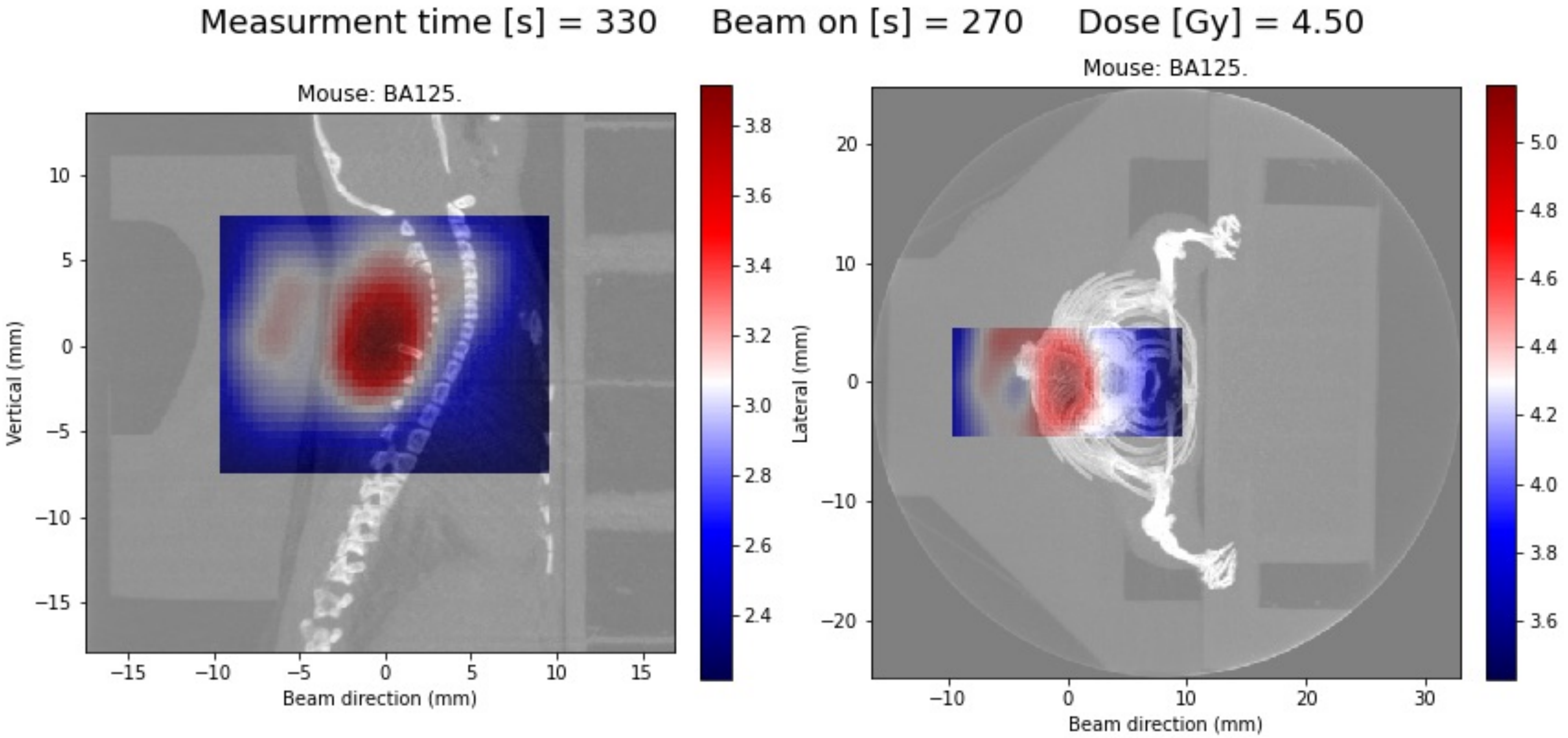
Measurment time [s] = 330 Beam on [s] = 270 Dose [Gy] = 4.50
Mouse: BA125.
Vertical (mm)
Beam direction (mm)
Mouse: BA125.
Lateral (mm)
Beam direction (mm)

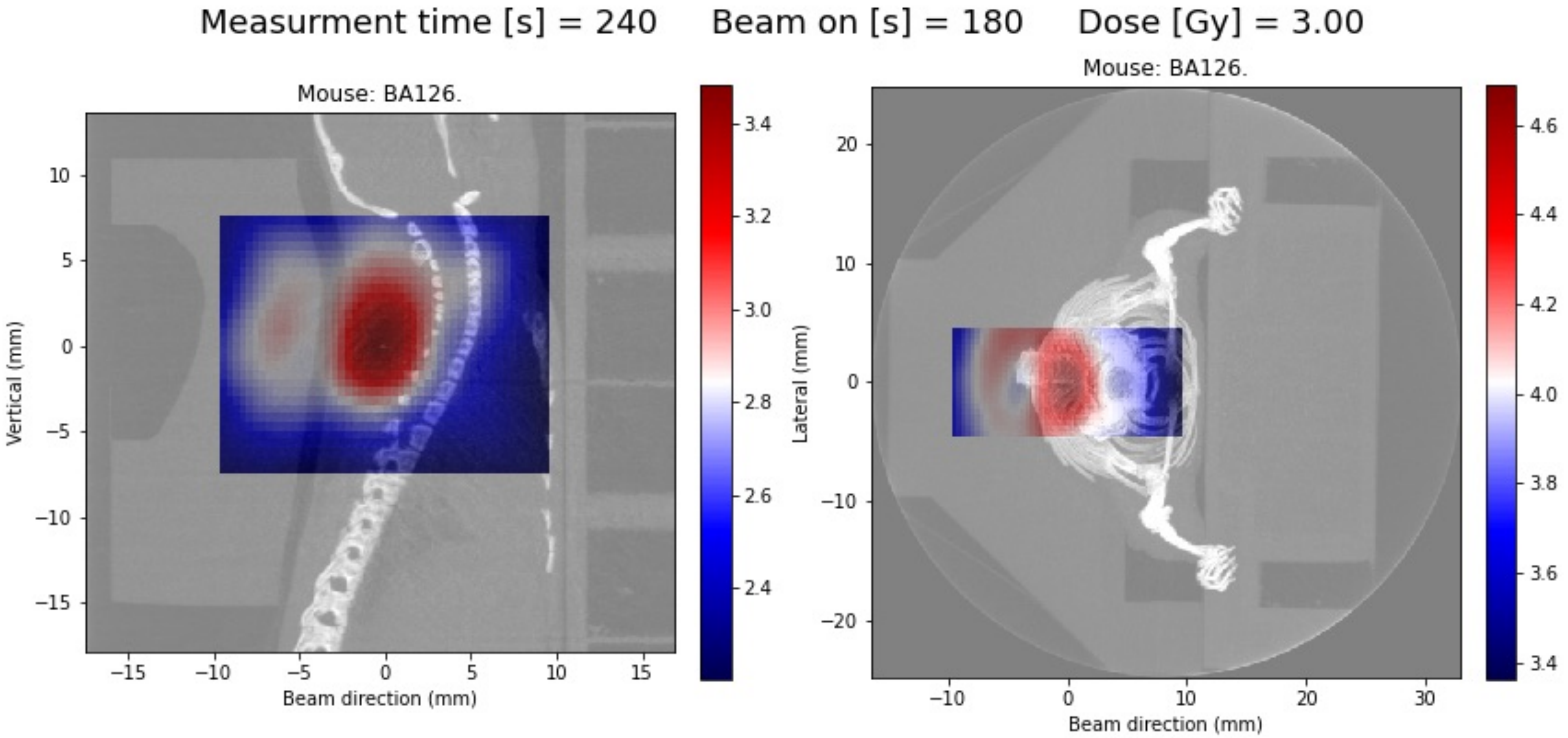
Measurment time [s] = 240 Beam on [s] = 180 Dose [Gy] = 3.00
Mouse: BA126.
Vertical (mm)
Beam direction (mm)
Mouse: BA126.
Lateral (mm)
Beam direction (mm)

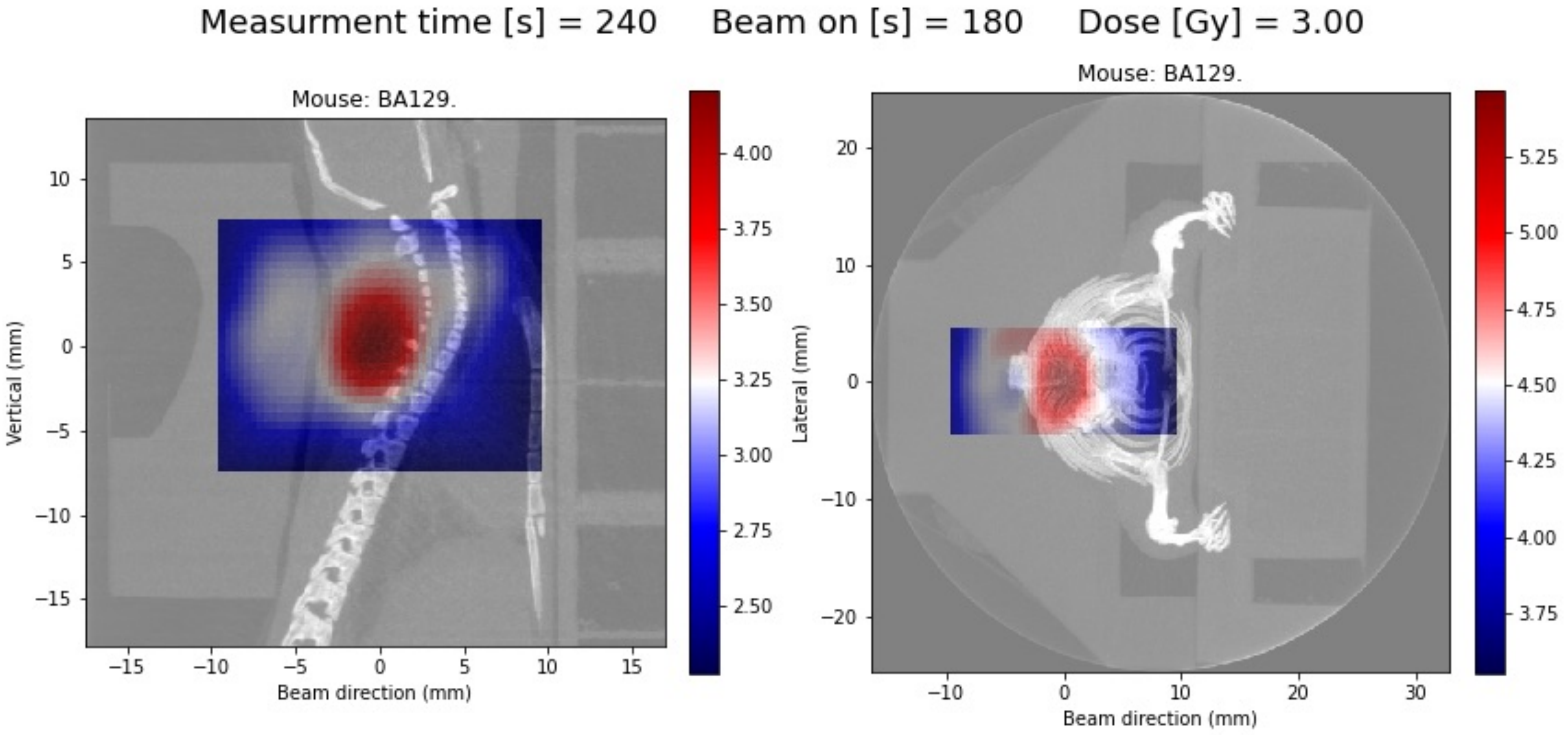
Measurment time [s] = 240 Beam on [s] = 180 Dose [Gy] = 3.00
Mouse: BA129.
Vertical (mm)
Beam direction (mm)
4.00
3.75
3.50
3.25
3.00
2.75
2.50
Mouse: BA129.
Lateral (mm)
Beam direction (mm)
5.25
5.00
4.75
4.50
4.25
4.00
3.75

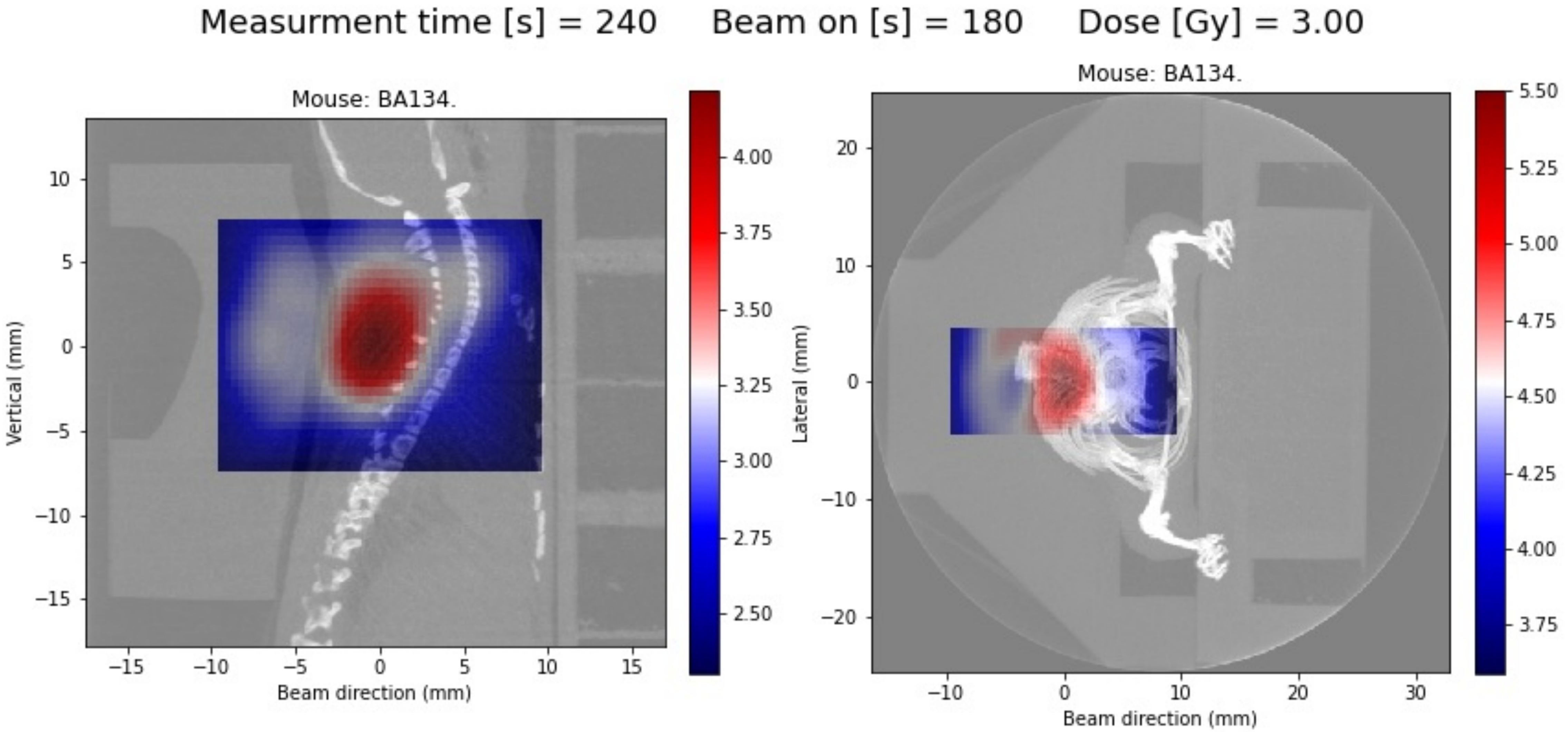

Measurment time [s] = 240 Beam on [s] = 180 Dose [Gy] = 3.00
Mouse: BA134.
Vertical (mm)
Beam direction (mm)
Mouse: BA134.
Lateral (mm)
Beam direction (mm)

Supplementary Fig. 2

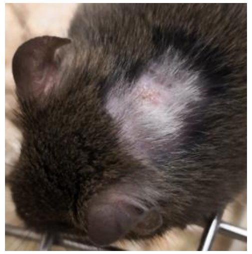

Grade 1

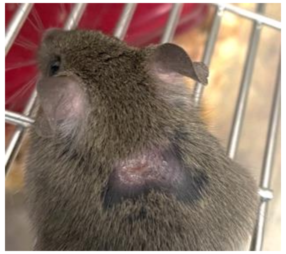

Grade 2

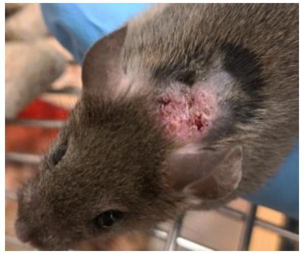

Grade 3

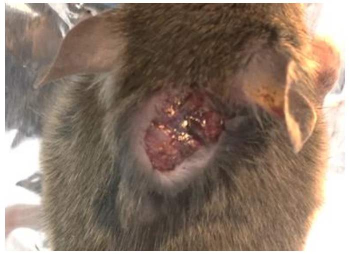

Grade 4

Grade 5	Necrosis
Grade 4	Open wound not healing, leading to sacrifice of the animal
Grade 3	Closed wound or small scratch
Grade 2	Dry skin, desquamation
Grade 1	Redness
Grade 0	No effect

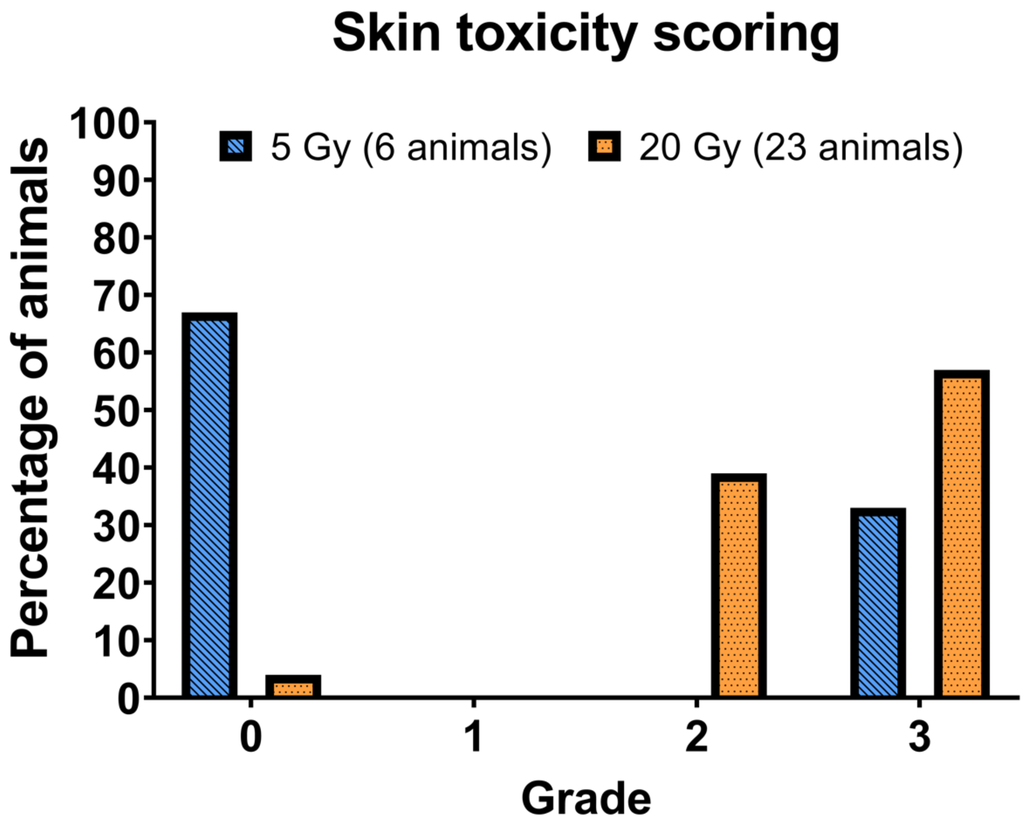

The Table shows the severity scale for the two different dose groups (5 Gy in light blue, n=6, 20 Gy in orange, n=23), and the images gives examples of the observed toxicities in the irradiated area. Grade 4 was only observed in control animals and was caused by excessive tumour growth. Those animals had to be sacrificed as specified in the ethical protocol. The plot shows the percentage of animals showing a certain toxicity grade, and has been created with GraphPad Prism version 10.5.0 (774).

Supplementary Figure 3. Grip strength test of individual mice. Grip test (Extended data Fig. 8) was performed every 2 weeks up to 6 months post-irradiation. **A.** Sham-irradiated mice (N=8) All data were used to calculate median grip performance and fraction of time points with F<100 N shown in Extended data Fig. 9. **B.** Irradiated mice (N=13). Here only data from week 6 were used to calculate median grip strength and fraction of time points with F<100 N, considering that myelopathy has a latency of at least one month. Every plot represents the data from one animal, and bars are standard deviations on three separate measurements for each mouse.

Supplementary Fig. 3A

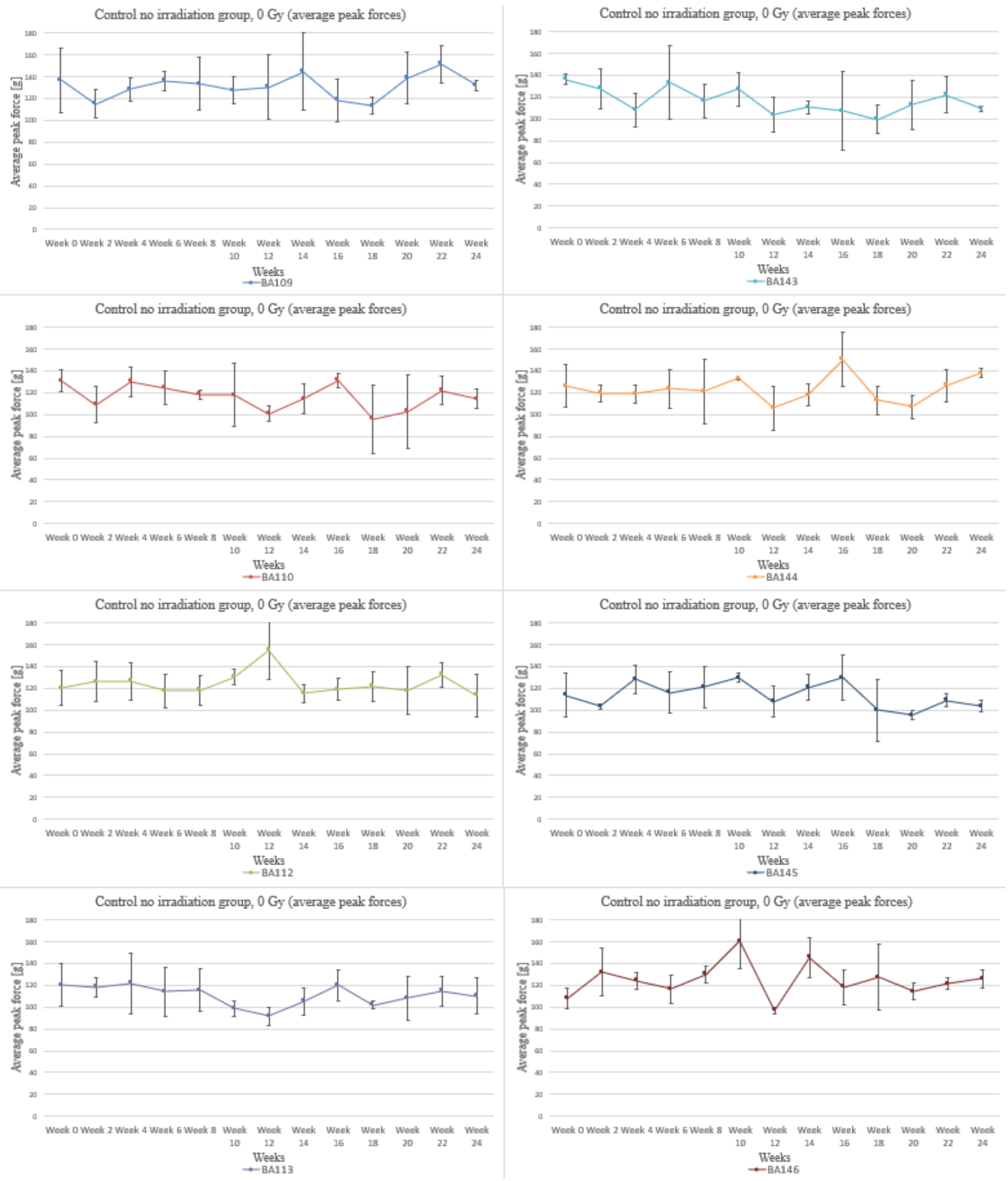

Single mouse plots 0 Gy

Supplementary Fig. 3B

Single mouse plots 20 Gy

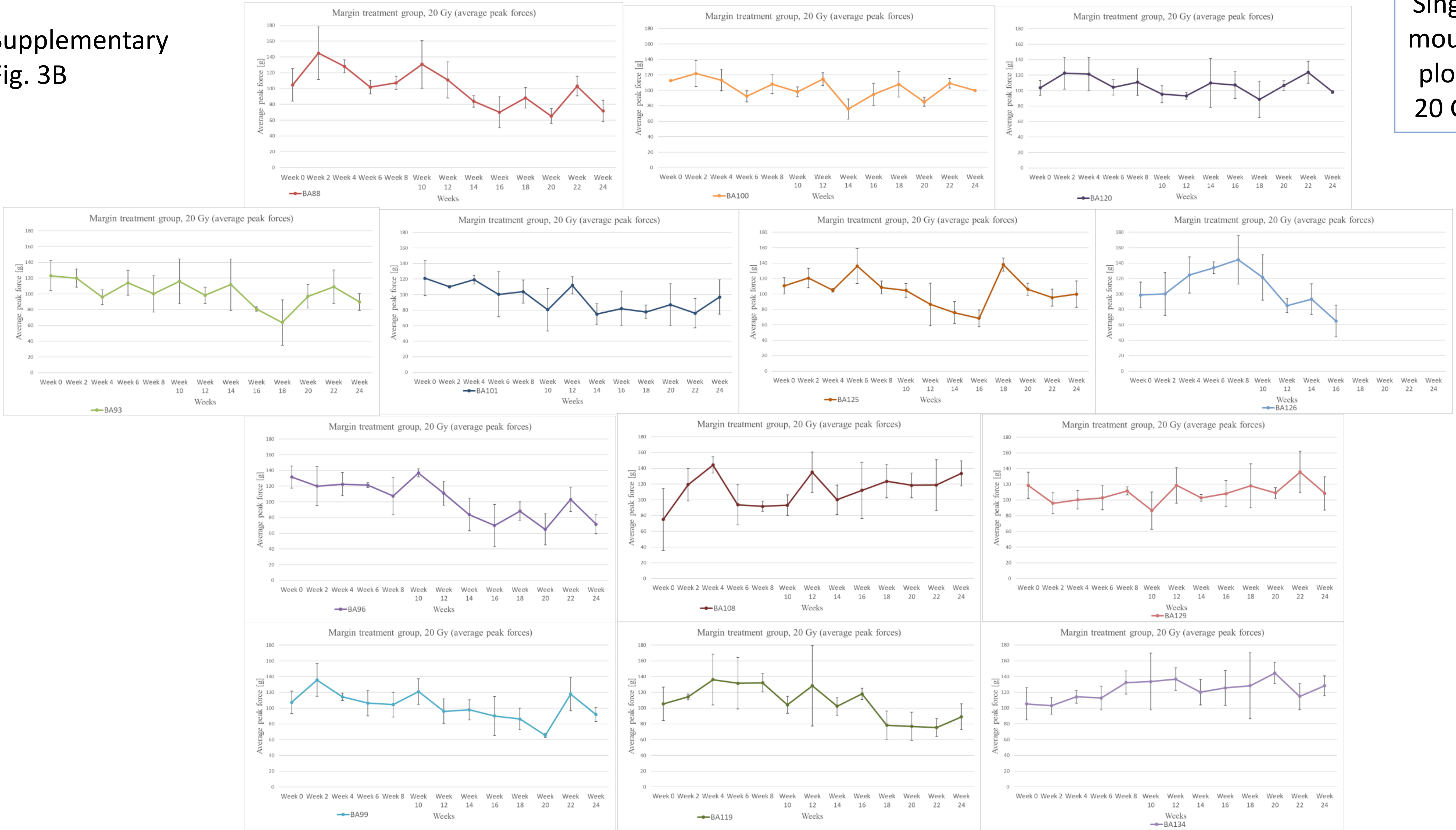

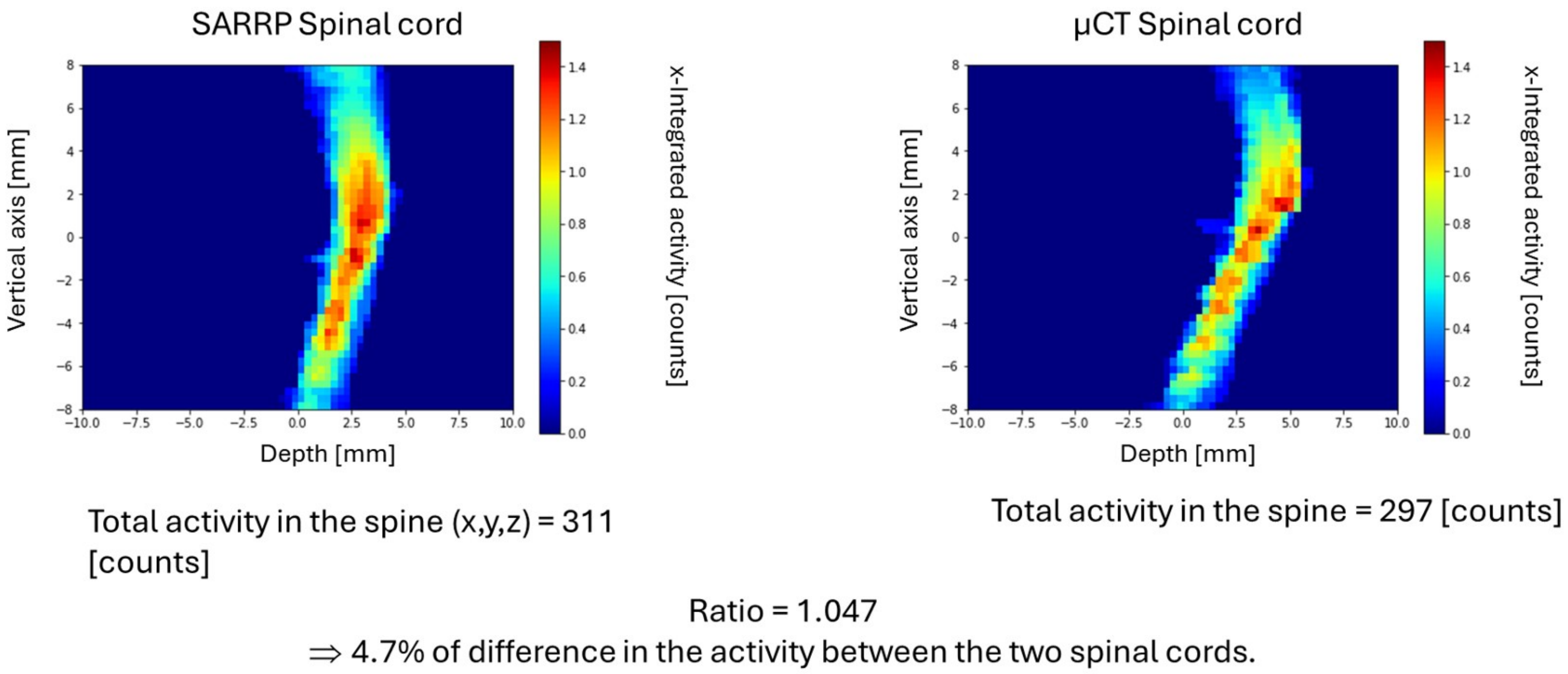

Supplementary Fig. 4. Activity in the spinal cord. A Monte Carlo simulation of the PET activity observed in the spinal cord using a horizontal mCT view or a vertical CBCT (SARRP). Images are reconstructed with the MLEM algorithm and the activity counts in the spine are normalized to the total activity in the image. The animals in vertical position always get slightly higher activity counts, but the value is in the range 2%-5%. These small differences do not affect the shape of the correlations shown in Extended Data Fig. 9 C-D, where the scatter is dominated by the high variability of the grip test results (Supplementary Fig. 3).

Supplementary Figure 5. Washout in individual mice. Washout data for all mice exposed to either 5 Gy (left pane) or 20 Gy ^{11}C-ions (right pane). Blue points represent the normalized activity decay measured in the mouse after the irradiation. Orange points represent the biological activity, i.e. activity normalized for the physical decay assuming the presence of 96% ^{11}C, 3% ^{11}C and 0.5% ^{15}O in the beam. The green curves correspond to the double-exponential fit of the biological activity decay (see Formula (1)). First, only the slow decay components were fit for time (t) values above 400 s to derive the k_s and the M_s parameters, and their values were utilized to fit the values of the fast decay component (k_f). One mouse from the 5 Gy group was excluded from the analysis because parameters were >3 σ far from the pooled data of the others.

Supplementary Fig. 5

5 Gy, double-exp fit, t_{long} = 400 s

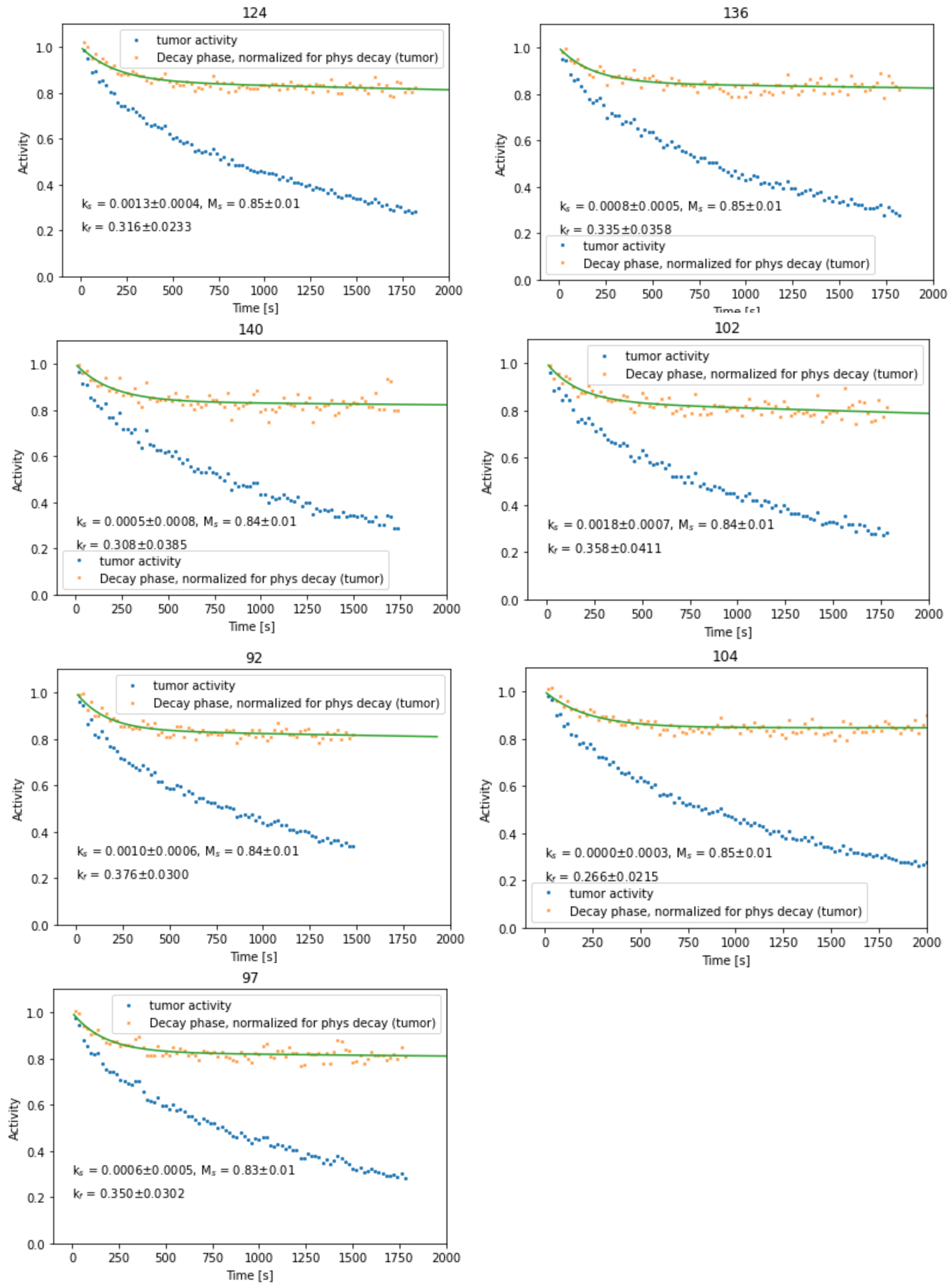

20 Gy, double-exp fit, t_{long} = 400 s

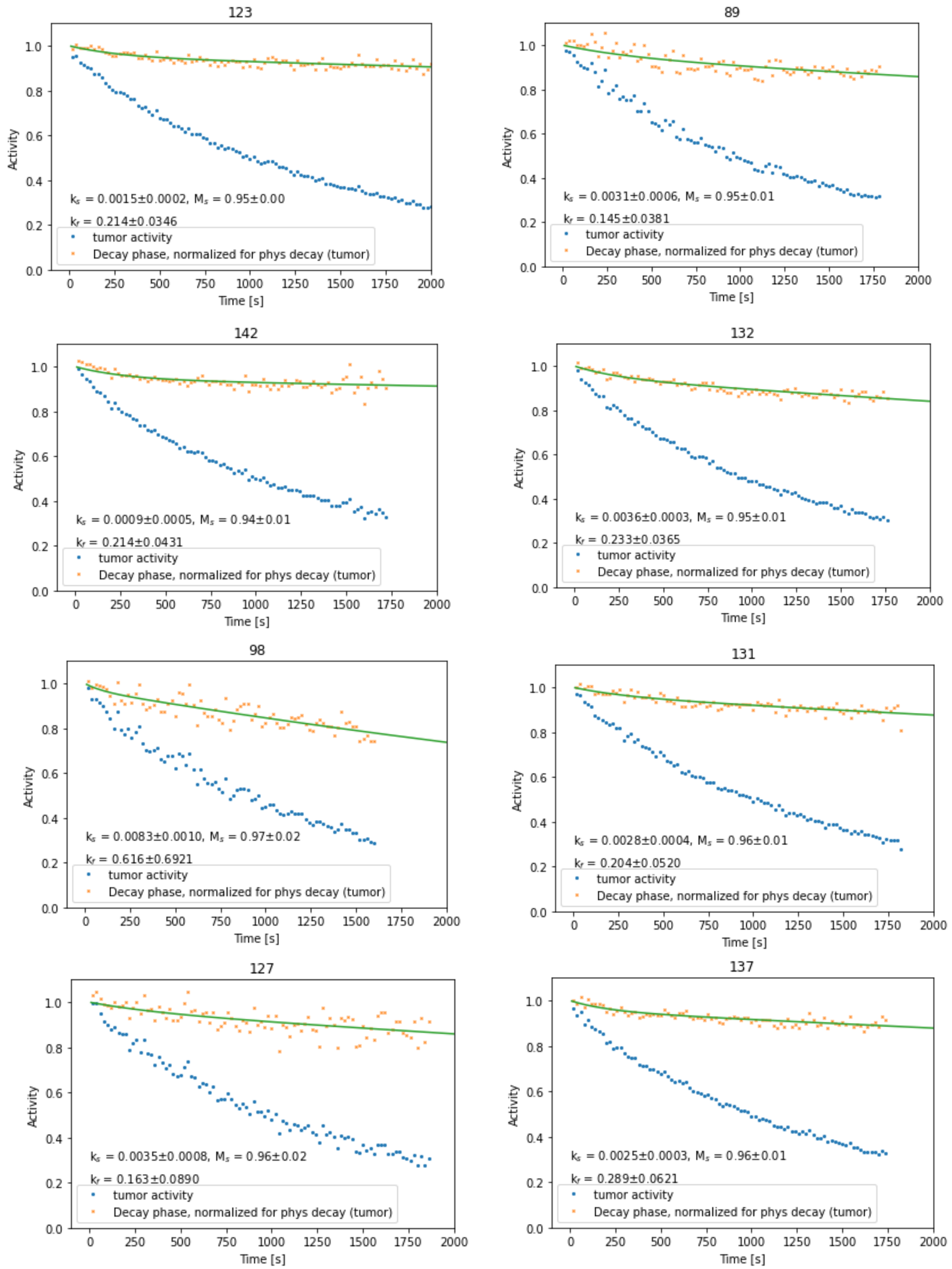

Supplementary Fig. 6

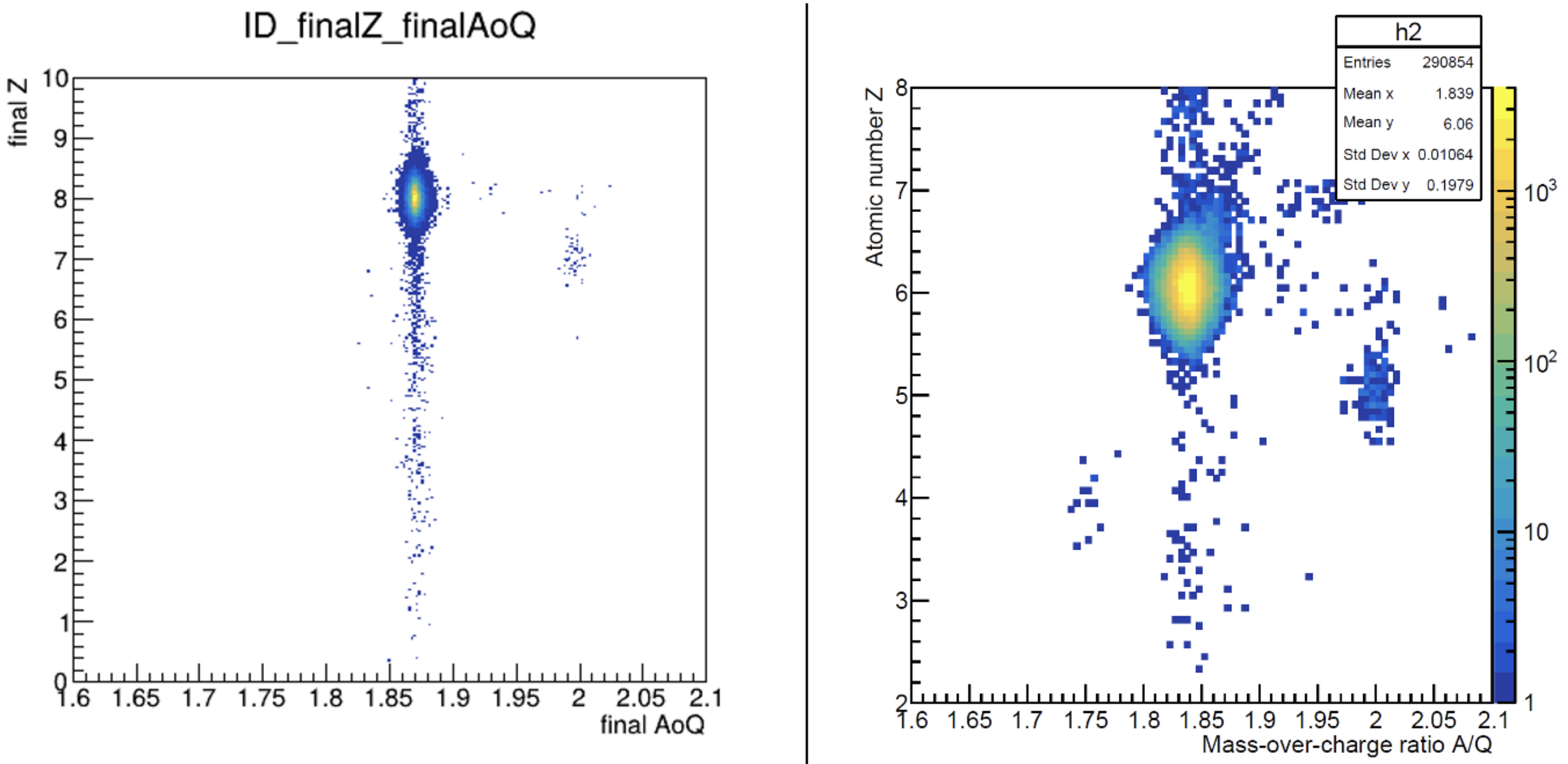

Beam purity (Left) Particle identification spectrum of ^{15}O, measured at the branch connecting the FRS and the medical cave. The parameters of the ^{16}O beam and settings of the separator were similar as during this experiment. The measured purity level more than 99%, the most prominent contaminations are ^{14}N. (Right) Particle identification spectrum of ^{11}C, measured at the main branch with similar parameters in terms of energy and production target as during this experiment. The purity level is 98%, the most prominent contaminations are ^{10}B and ^{7}Be.

SIPPLMENTARY MATERIAL: DOSIMETRY

1. Monte Carlo simulations of the beam profile along the experimental beamline

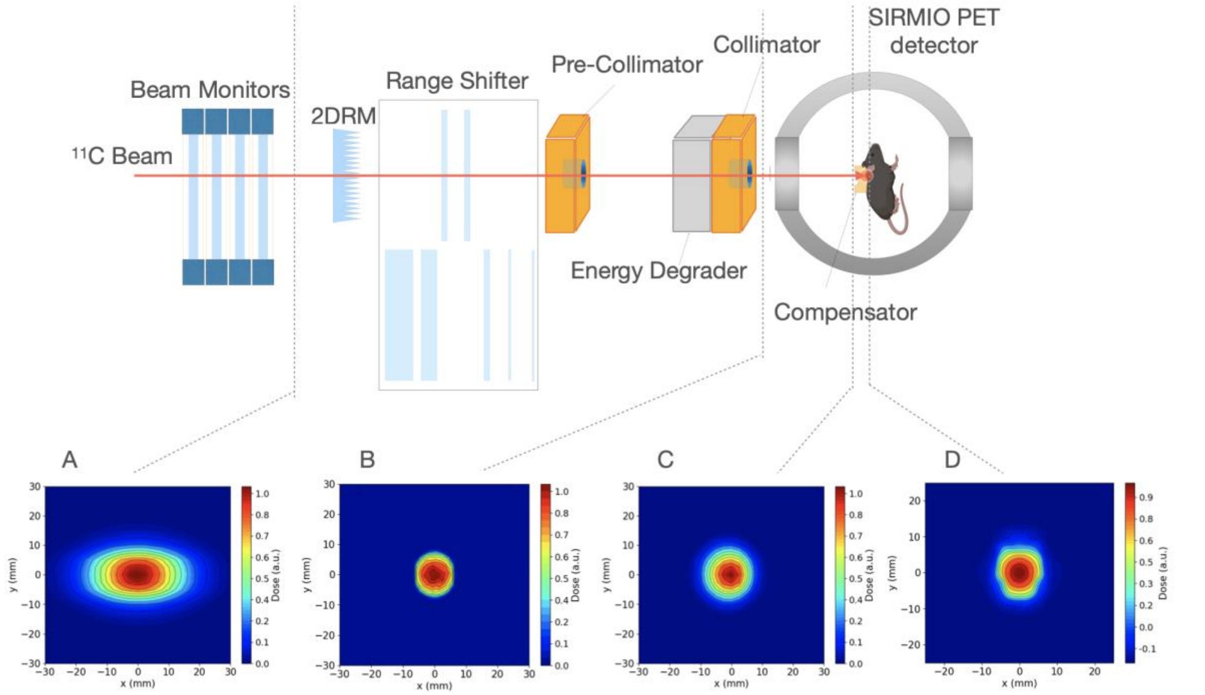

Supplementary figure 7: Schematic representation of the experimental beamline used for the animal irradiation. Panel A-D show FLUKA simulations of the lateral dose distribution at different position along the beamline, respectively: A) at the entrance in the experimental room after the beam monitor detectors, B) at the collimators, C) at the mouse collar, D) at the tumor position.

Supplementary Figure 7 presents a schematic representation of the experimental setup used for animal irradiation. To illustrate the function of the different components, the simulated lateral dose distribution at various positions along the beamline is also shown. The beam parameters used in these simulations were obtained during beam characterization measurements, as described in the following sections.

From left to right, the beamline consists of the following elements:

- **Beam Monitoring:** The pristine ^{11}C beam enters the experimental room and is monitored using standard large-plate ionization chambers installed in the nozzle of the GSI medical cave. These chambers, calibrated in terms of the number of primary particles, are integrated with the dose delivery system. Since the ^{11}C beam is a secondary beam, its phase space is larger than that of a typical ^{12}C pencil beam used in particle therapy, resulting in a larger beam spot size and momentum spread. Measured values are reported in Extended Data Table 1. A simulated 2D dose distribution of the beam at the entrance of the experimental room is shown in Figure S1 Panel A.
- **2DRM for SOBP Energy Modulation:** A 2D range modulator (RM) was used to shape the beam energy, creating a 1.2 cm spread-out Bragg peak (SOBP) in water. This SOBP fully covers the clinical target volume (CTV) while allowing some margin for range adjustments during treatment.

- **Range Shifter and Aluminum degraders for range adjustment:** Aluminum plates with a total thickness of 28.4 mm (equivalent to 60.1 mm of water path length) were introduced into the beamline as bolus material. These plates shifted the beam range to approximately match the tumor position. A range shifter equipped with movable PMMA plates was used to fine-tune the distal fall-off of the SOBP in the mouse, with a resolution of 0.5 mm water-equivalent thickness. The range shifter plates and aluminum thickness were chosen to achieve a residual beam range of 1.32 cm in water, corresponding to 0.56 cm in the mouse neck (after accounting for the mouse collar/compensator). The water-equivalent thicknesses of all degradation materials were characterized in the beam before the animal experiment.
- **Collimation:** A series of brass collimators was used to reduce the lateral irradiation field and blocked parts of the beam that did not contribute to the target dose. This was done to improve the signal to noise ratio and limit the amount of stray radiation on the SIRMIO PET scanner. To reduce as much as possible the effects of lateral scattering, the collimators were placed as close as possible to the aperture of the SIRMIO PET scanner. The collimator aperture consisted in an ellipse with the horizontal axis of 1.2 mm and the vertical one of 1.5 mm. To uniformly cover the collimator aperture, the final irradiation plan used two pencil beams with a 6 mm shift in the y-direction, delivered over 20 rescans to improve uniformity. A simulated lateral dose distribution of the collimated beam at the exit point from the collimator and at the surface of the mouse collar is shown in Figure S1B and Figure S1C respectively.
- **Compensator:** Finally, a plastic mouse collar, acting as a compensator, was fixed onto the mouse bed. This component was designed to partially absorb the beam outside the CTV and shape the distal edge of the SOBP to match the target contour. The expected lateral dose distribution at the center of the CTV (corresponding to the isocenter of the experimental room) is illustrated in the third panel of Figure S1D.

2. ^{11}C beam characterization and depth dose measurements in water

As described in the *Materials and Methods* section, both 1D and 3D dose measurements of the pristine and range modulated ^{11}C beam were acquired in water using two setups: a PEAKFINDER™ system (PTW Freiburg, Germany) for the 1D depth dose distribution and a water phantom setup equipped with a PTW OCTAVIUS 1600 XDR detector for 3D dose maps. A schematic representation of the water phantom setup is shown in Supplementary Figure 8. The entrance window of the water phantom was positioned at the isocenter, and no attenuator or beam-shaping device—other than the 2DRM for SOBP generation—was introduced into the beamline.

This setup allowed for the measurement of 2D dose maps at different positions in water, starting from a minimum water-equivalent thickness of 2.10 ± 0.05 cm. These maps were then merged to create a full 3D dose distribution with an accuracy on the relative dose better than 2%. Supplementary Figure 9 and 10 present a comparison of these measurements with Monte Carlo simulations for both the pristine and SOBP configurations. Additionally, lateral dose profiles at selected water depths are compared.

The measured data were essential for extracting key beam parameters, including beam range in water, beam energy, beam spot size, and momentum spread. This information was subsequently used to determine the exact absorber thickness required to completely stop the beam at the distal end of the mouse CTV and to define the beam model for Monte Carlo simulations (see Extended Data Table 1).

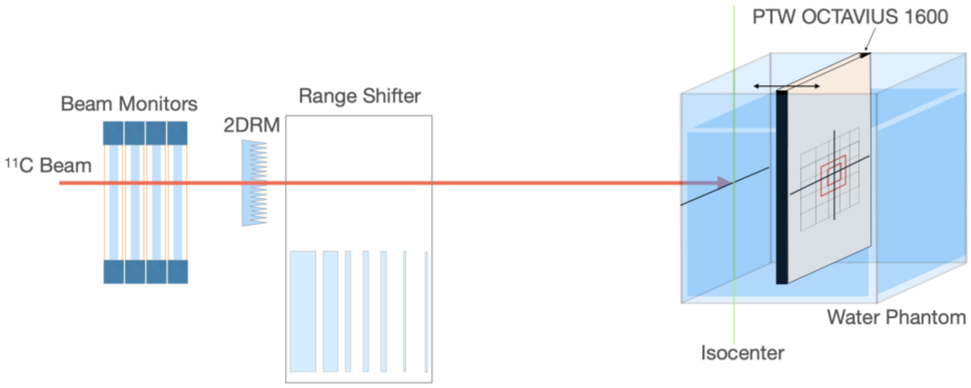

Supplementary Figure 8: Schematic representation of the water phantom experimental setup used for 3D dose measurements and beam characterization.

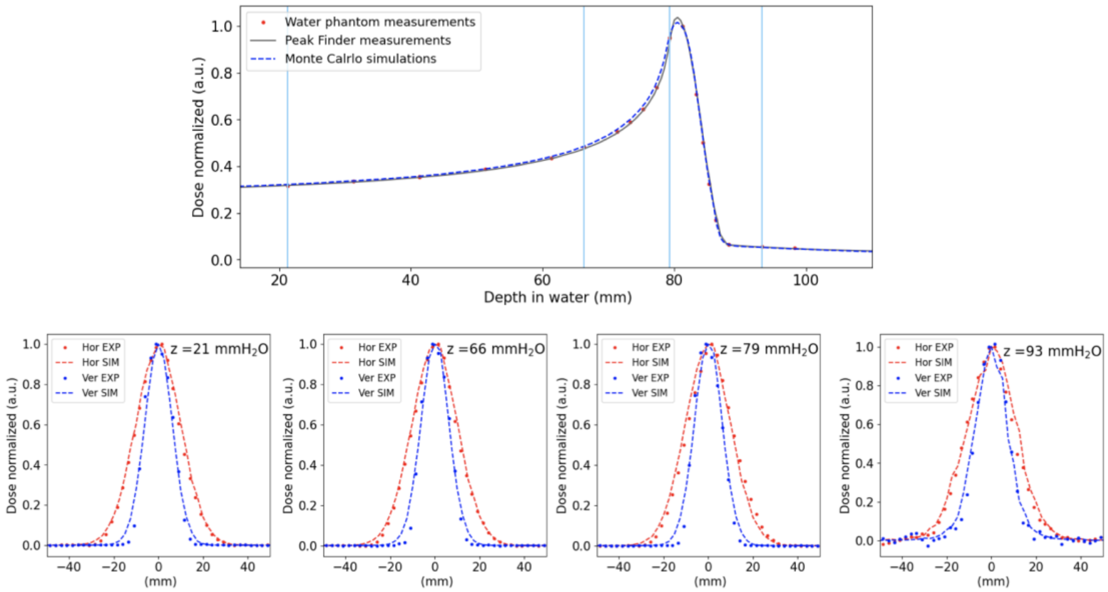

Supplementary Figure 9: Measured laterally integrated depth dose distribution for the pristine beam and respective Monte Carlo simulations (upper panel). In the lower panel comparison of the measured lateral dose distribution with the corresponding Monte Carlo simulation at selected depth in water (represented with light blue lines on the depth dose profile).

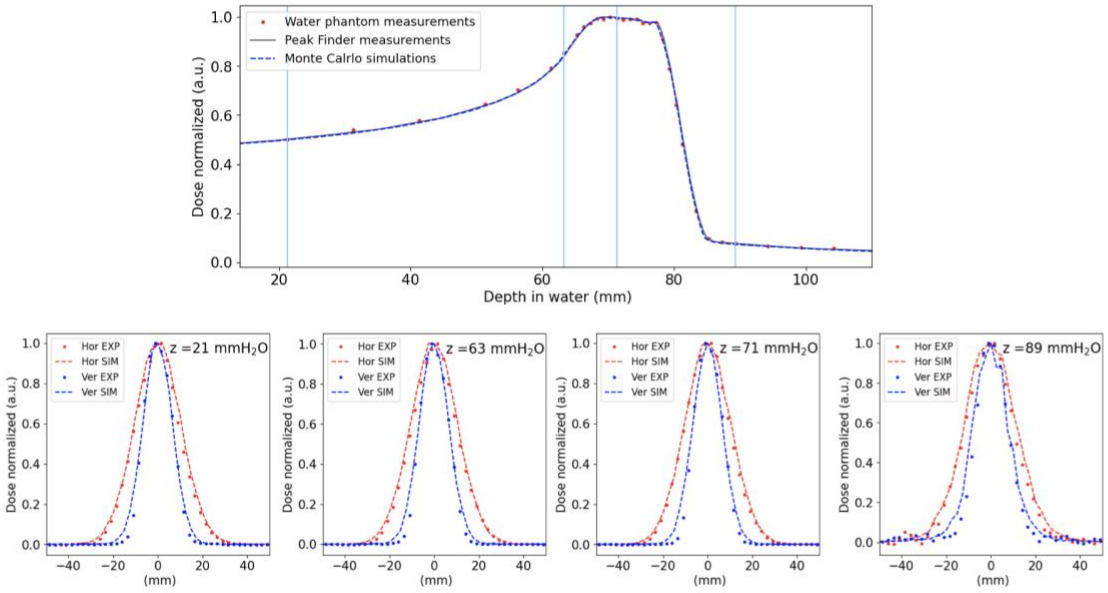

Supplementary Figure 10: Measured laterally integrated depth dose distribution for the SOBP beam configuration and respective Monte Carlo simulations (upper panel). In the lower panel comparison of the measured lateral dose distribution with the corresponding Monte Carlo simulation at selected depth in water (represented with light blue lines on the depth dose profile).

3. Beam characterization and dosimetry at the mouse position

3.1. Lateral dose distribution measurements at the target positions

Once the beamline for the animal irradiation was defined and built, the 2D dose distribution of the beam arriving at the target position was measured with OCTAVIUS 1600 XDR detector free in air (Supplementary Figure 11). In this case it was not possible to use the water phantom, since the residual range in water at this stage was smaller than the minimal measurable water thickness. Supplementary Figure 12 shows the measured 2D dose distribution and a comparison of the lateral dose profiles along the central lateral and horizontal axis with the corresponding Monte Carlo simulations.

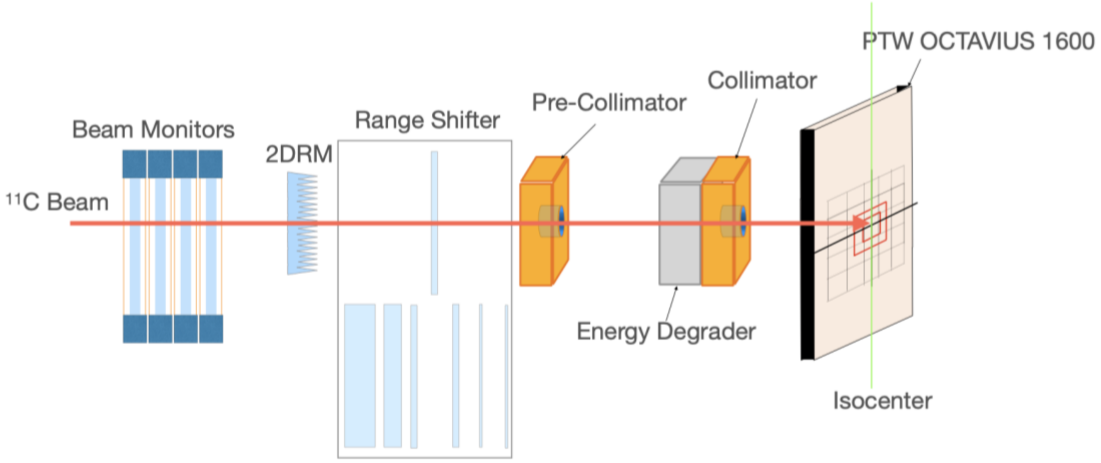

Supplementary Figure 11: Schematic representation of the experimental set up used to verify the dose distribution at the target position with the set up for the mice irradiation on the beamline.

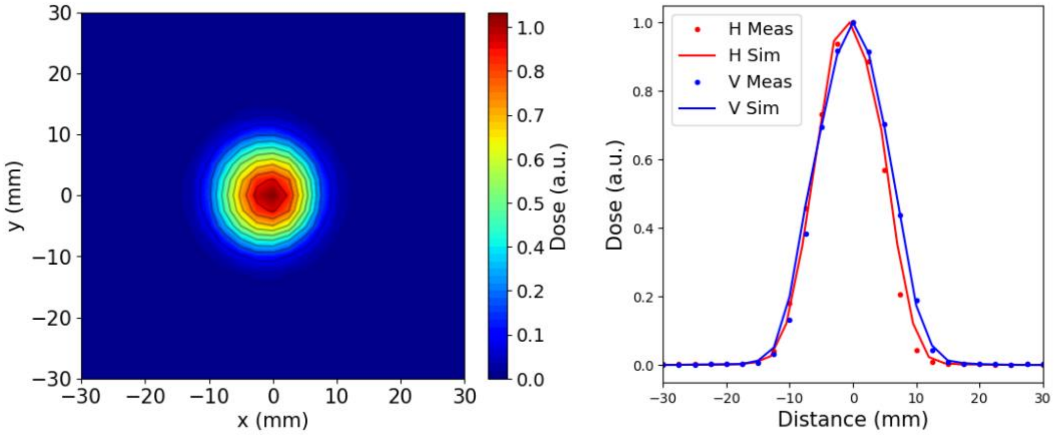

Supplementary Figure 12: Panel A, measured 2D dose distribution after beam energy degradation and collimation as it was then adopted for the animal experiment. Panel B, a comparison of measured and simulated lateral dose profiles at the target position after the beamline to be used for the animal irradiation was set up.

3.2. Dosimetric calibration at the tumor position

A final dosimetric calibration was performed at the tumor position using a volume ionization chamber (PTW TM31023), as shown in Supplementary Figure 13. A custom-designed compensator, shaped like a mouse collar, was used to secure the chamber in a fixed position at the center of the CTV (Supplementary Figure 14). This measurement enabled us to establish a monitor unit calibration relative to the dose at the target position, which was then used to adjust the irradiation plan and ensure the accurate delivery of the desired dose to the target. The chamber readings were recorded using a UNIDOS electrometer, applying the standard correction factors commonly used in particle therapy. Specifically, a k_Q factor of 1.03 was used, along with a k_{TP} correction based on the measured temperature and pressure inside the irradiation room during the experiment. The k_S factor was neglected due to the low dose rates.

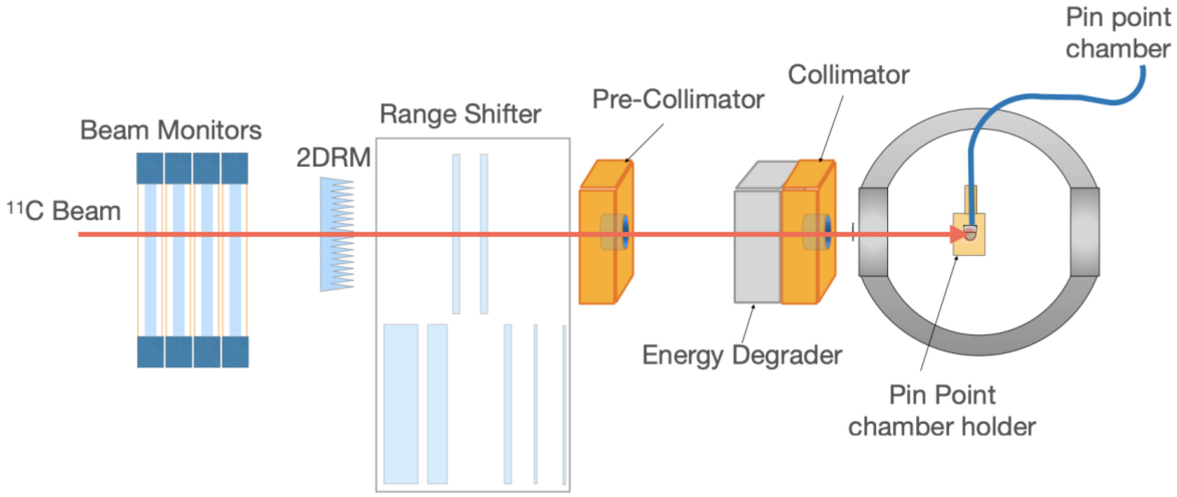

Supplementary Figure 13: Schematic representation of the experimental set up used for absolute dosimetry at the tumor position

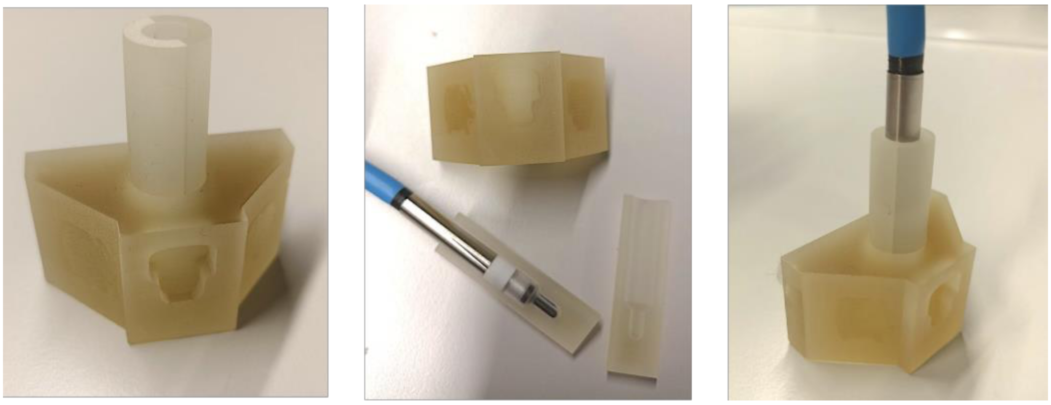

Supplementary Figure 14: Pictures of the pin point chamber holder designed for dose calibration at the tumor position. The holder shape resemble the mouse compensator and has an insert for a PTW pin point chamber holding the chamber at a position and a water equivalent depth corresponding to the center of the CTV. The holder was attached to the mouse bed in the same position as a the mouse compensator.
